# Coniferyl ferulate alleviate xylene-caused hematopoietic stem and progenitor cell toxicity by Mgst2

**DOI:** 10.3389/fphar.2024.1334445

**Published:** 2024-03-08

**Authors:** Zhao Yin, Ruiming Ou, Yangmin Zhu, Zhi Liu, Jing Huang, Qi Zhong, Guangchao Li, Qing Zhang, Shuang Liu

**Affiliations:** Department of Hematology, Guangdong Second Provincial General Hospital, Jinan University, Guangzhou, China

**Keywords:** xylene, HSPCs, mgst2, coniferyl ferulate, hematotoxic

## Abstract

Xylene exposure is known to induce toxicity in hematopoietic stem and progenitor cells (HSPCs), leading to bone marrow suppression and potential leukemogenesis. However, research on the gene expression profiles associated with xylene-induced toxicity in HSPCs, and effective therapeutic interventions, remains scarce. In our study, we employed single-cell RNA sequencing to capture the transcriptomic shifts within bone marrow HSPCs both prior to and following treatment with coniferyl ferulate (CF) in a mouse model of xylene-induced hematotoxicity. Subsequently, we pinpointed CF as a targeted agent using SPR-LC/MS analysis. This enabled us to confirm the link between the gene Mgst2 and specific cellular subtypes. Our data revealed that CF significantly countered the reduction of both monocyte and neutrophil progenitor cells, which are commonly affected by xylene toxicity. Through targeted analysis, we identified Mgst2 as a direct molecular target of CF. Notably, Mgst2 is preferentially expressed in neutrophil progenitor cells and is implicated in mitochondrial metabolic processes. By selectively inhibiting Mgst2 in bone marrow, we observed amelioration of xylene-induced hematotoxic effects. In summary, our findings suggest that coniferyl ferulate can mitigate the detrimental impact of xylene on hematopoietic stem and progenitor cells by targeting Mgst2, particularly within subpopulations of neutrophil progenitors. This discovery not only advances our comprehension of the cellular response of HSPCs to xenobiotic stressors like xylene but also identifies CF and Mgst2 as potential therapeutic targets for alleviating xylene-induced hematotoxicity.

## Introduction

The air around humans is contaminated by several toxic chemicals, which might cause severe disorders (Hassan and Aly, 2018; Inesta-Vaquera et al., 2023). These contaminants might enter the body via breathing and cause harm (Kang et al., 2021). Typically, aromatic compounds, such as benzene, xylene, and toluene are toxic to humans (Snyder, 2012). A threshold limit value (TLV) of 100 ppm in the working environment is recommended by the health and safety authorities of many nations, including China. Around 95.2% of the workforce showed cumulative carcinogenic risk due to the inhalation of xylene. Also, around 99.9% of the distribution of carcinogenic risk is attributed to the benzene metabolite N-acetyl-S-(phenyl)-L-cysteine of over 10^–6^ (Sun et al., 2022). Benzene is a well-known hemotoxin, which is classified by the International Agency for Research on Cancer as a category 1 carcinogen (Lan et al., 2004; Mosmeri et al., 2019). However, no effective treatment is known that specifically alleviates the hematotoxicity caused by xylene. Therefore, the mechanisms need to be elucidated, and the control over xylene -caused hematotoxicity needs to be optimized for developing new treatments.

Exposure to xylene can cause injury to the hematopoietic system, such as different degrees of pancytopenia, aplastic anemia, and even leukemia (Mokammel et al., 2022; Vermeulen et al., 2023). Leukemia caused by chronic exposure to benzene is a significant public health issue that has raised concerns among health system worldwide. Exposure to a low dose of benzene (≤1 ppm) can induce injury to the hematopoietic system (Wang et al., 2022). Many studies on toxicology and epidemiology have investigated the mechanism by which benzene increases toxicity in the blood, but the precise mechanism needs to be elucidated. Thus, a comprehensive investigation of the mechanism by which xylene triggers hematological injuries is required. In a conventional hierarchical model, HSCs exhibit sequential differentiation first into multipotent progenitors (MPPs) and then into common lymphoid progenitors (CLPs), as well as, myeloid progenitors (CMPs). Later, they produce granulocyte-macrophage progenitors (GMPs) along with megakaryocyte-erythroid progenitors (MEPs) (Crippa et al., 2023). However, traditional bulk expression analysis of heterogeneous populations can be performed to assess the mean expression levels of all cells in the sample, which does not represent the expression level in every cell (Yin et al., 2023). The single-cell profiling technology can resolve the problem of population heterogeneity (Xia et al., 2023). Therefore, single-cell transcriptome sequencing technology was used in this study to elucidate the mechanism by which xylene causes damage to hematopoietic cells.

Salimi *et al.* examined the effect of xylene on inducing ROS production and impairment of the mitochondria in lymphocytes (Salimi et al., 2017). Xylene causes the accumulation of reactive oxygen species (ROS) and oxidative stress in yeast cells (Singh et al., 2010). Nishida-Aoki *et al.* showed that the organic solvent isooctane can promote ROS production in yeast cells by impairing mitochondria. Similarly, xylene may also impair mitochondria, resulting in the release of ROS along with mitochondrial fragmentation in yeast cells (Nishida-Aoki et al., 2015). This hypothesis might be reasonable, considering that xylene can impair mitochondria in human cells and induce mitochondrial morphological alterations in yeast cells. However, the key molecular mechanism by which xylene induces ROS accumulation needs to be determined.

Angiotensin II-induced leukotriene C4 (LTC4) can induce ROS accumulation, and it is exclusively expressed in hematopoietic cells, such as mast cells (Fujimori et al., 2022). Additionally, microsomal glutathione S-transferase 2 (Mgst2), the isoenzyme of LTC4, is ubiquitously expressed in non-hematopoietic cells and has different functions (Thulasingam et al., 2021). The Mgst2-LTC4 signaling cascade can be activated by common chemotherapeutic agents and ER-mediated stress. It induces oxidative stress, oxidative DNA damage, and ROS-induced cell death (Dvash et al., 2015).

Coniferyl ferulate is a phenolic acid compound abundant in umbelliferae plants with multiple pharmacological activities. It has many pharmacological effects, such as antioxidation, antibacterial effects, and vasodilation (Gong et al., 2020). CF is widely used for enriching and nourishing blood and improving blood circulation. Some studies have found that ferulic acid, an analog of CF, can increase blood fluidity, decrease serum lipids, suppress platelet aggregation, prevent thrombogenesis, protect neurons such as PC12 cells, and exhibit potent antioxidant effects (Gong et al., 2020). However, the relationship between the pharmacological effects of CF on xylene and damage to the hematopoietic system needs to be elucidated.

In this study, we investigated whether CF supplementation can protect mice against xylene-caused damage to the hematopoietic system and the mechanism by which CF exerts its effects at single-cell resolution. Additionally, the direct target of CF was also assessed. CF can directly bind to Mgst2, it can alleviate the damage to the hematopoietic system caused by xylene. The subcluster of neutrophil progenitors and monocyte progenitors are considered to be the real responsive cells in HSPCs. Mgst2 is the potential therapeutic target for treating xylene toxicity, and CF is a therapeutic agent that can alleviate the toxic effects of xylene.

## Materials and methods

### Animals

Normal male C57BL/6 SPF mice [n = 45, 8 weeks old, and 20 ± 2 g; SYXK (Yue) 2017–0174] were obtained and adaptively fed for 7 days before the experiment. All mice were maintained under experimental conditions of 21°C ± 2°C, 30%–40% relative humidity, and a 12-h/12-h light/dark cycle. All animal experiments were approved by the Animal Ethics Committee (IACUC-20220429–01) and were conducted following the animal-welfare guidelines. All animals were randomly placed into three groups, including the control, xylene (150 mg/kg), and xylene (150 mg/kg) + CF (50 mg/kg) groups.

### Drug preparation and intervention

Coniferyl ferulate (CF) (purity: ≥98%; high-performance liquid chromatography grade) was purchased from Chengdu RefMedic Biotech Co., Ltd. (Chengdu, China). All mice in the CF group were administered 50 mg/kg CF once a day through intragastric administration. CF (50 mg) was dissolved in 0.1% ethanol to prepare a CF solution at a final concentration of 10 mg/mL.

The mice in the other two groups were administered 1X PBS (Phosphate-Buffered Saline) supplemented in 0.1% ethanol. Two weeks before exposure to xylene, CF or PBS was orally administered to the mice.

### FCM analysis

To analyze stem cells, we harvested mouse bone marrow cells (BMCs) from the six-week-old C57BL/6 mice and stained them. After extraction, BMCs were lysed with red blood cells (RBCs) and incubated with several antibodies. Then, we prepared splenic tissue and BMC-derived single-cell suspensions and stained them for 20 min on ice. The HSPC markers applied included LT-HSC: Lin- (PerCP-Cy™5.5); FVS780- (APC-Cy™7); C-KIT+ (CD117, APC); Sca+ (PE-Cy™7); CD34^−^ (FITC); Flk2- (BV421); GMP: Lin- FVS780- C-KIT + Sca- CD34^+^ CD16/32+ (FcγRII, BV421); MPP: Lin- FVS780- C-KIT + Sca+ CD34^+^ Flk2+; MEP: Lin- FVS780- C-KIT + Sca- CD34^−^ CD16/32-; CMP: Lin- FVS780- C-KIT + Sca- CD34^+^ CD16/32-; GR-1 (Ly-6G and Ly-6C), and MAC-1(CD11b); B cell (CD19); T cell (CD90.2). The antibodies used for FCM were purchased from BD Biosciences (New Jersey, United States of America). FACSVerse (Becton Dickinson) was used for examining cells. The FCM data were analyzed using the FlowJo software (TreeStar, Inc.).

### Colony-formation assay

We analyzed methylcellulose colony formation using 100,000 mouse HSPCs. Specifically, the mouse MethoCult medium (StemCell Technologies) was used for preparing the cell suspension. Then, cell culture was performed using culture plates (3 cm in diameter), and evaporation was prevented through the addition of excessive PBS. The number of colonies formed was counted after 14 days.

### Single-cell RNA sequencing

Bone marrow cells (BMCs) were harvested from C57BL/6 mice placed in different groups. First, using the Lineage Cell Depletion Kit (mouse), CD117 microbeads (Miltenyi Biotec) were used to obtain CD117+ cells. Additionally, samples were prepared with the 10x Genomics Single Cell 3′v2 Reagent Kit following the manufacturer’s protocol. About 6,000 cells were collected from each sample for constructing the cDNA library on a 10x Genomics single-cell-A chip following the protocol provided with the Single Cell 3′Reagent Kit v3. The PCR cycle program was slightly modified based on the expression of cDNA (recommended by 10X Genomics). The samples were pooled, normalized to 10 nM, and diluted to 2 nM using an elution buffer containing 0.1% Tween 20 (Sigma). Then, the samples were sequenced to a median depth of 50,000 reads/cell with the Novaseq 6000 system using the parameters below: read 1–26 cycles, read 2–98 cycles, and index one to eight cycles.

### Single-cell trajectory analysis

The Monocle 2 algorithm (v2.8.0) of R.18 was used to generate HSPC trajectories. Briefly, using the CellDataSet function, the CellDataSet object was created using raw UMI counts under default parameters. Those genes with an average level of <0.1 were eliminated from the trajectory analysis, whereas the DEGs that satisfied a q-value of <0.01 in 2 cell groups were implemented in dimension reduction using the reduce Dimension() function, with the following parameters, max_components = 2 and reduction method = ‘DDR_Tree’. Next, we performed single-cell ordering and visualization using the plot_cell_trajectory() function, followed by coloring according to pseudo time or cell groups. The genes that showed differential levels between branches were identified using the branch expression analysis modeling (BEAM) algorithm, whereas those that satisfied a q-value of <10^–10^ were placed in different clusters, followed by visualization using the plot_genes_branched_heatmap() function. The clusterProfiler (version 3.14.3) package in the R software was used for GO functional annotation of the genes in different clusters.

### Interactions between cell types

Using the CellChat algorithm (version 0.0.2; https://github.com/sqjin/CellChat), the ligand-receptor (L-R) interactions were analyzed based on the ligand levels within a type of cell, as well as the respective receptors in another type of cell. Permutation tests were conducted to identify significant L-R interactions (*p* < 0.05). Bubble plots were constructed to visualize the L-R interactions in various cell sub-clusters. Additionally, the L-R interactions, along with their associated strengths, were compared among different cell populations, and then they were visualized using heat maps.

### Gene set variation analysis (GSVA)

We obtained gene sets based on the Cancer Single-cell State Atlas (CancerSEA) and the Molecular Signature Database (MSigDB). Using the GSVA function of the GSVA package (version 1.38.2), the pathway scores of the cells were determined, whereas the limma R software package (version 3.46.0) was used for assessing differential pathways.

### Identification of targets based on surface plasmon resonance-high performance liquid chromatography-mass spectrometry (SPR-HPLC-MS)

Initially, after CF was fixed on the 3D optical cross-linking chip (Photo-cross-linking Sensor Chip), CD117+ cell lysates were circulated on the chip surface. The SPR technology was used for real-time monitoring of the process in which the chip surface molecules captured target proteins. The CF-captured target proteins were determined with the LC-MS consortium, whereas the bioinformatics database was used for functional analysis.

To quantitatively calibrate CF, first, 5 mM CF was prepared using a DMSO solution. Next, array printing was conducted using the BioDot™ −1520 array printer. For maintaining the sample quantity point, we used a point diameter of 180 µm and a point spacing of 280 µm. In this study, a double-needle printing system was used and consisted of the chip surface that contained a 5050 dot array, point solution (2.5 nL), repeated point samples (10 folds), and chip surface point sample (31.25 µL, 312.5 nMol).

Using the protein quantification kit (Thermo Fisher, BCA Protein Assay Kit), cell lysis was performed at a concentration of 498.15 μg/mL. The concentration of the sample was adjusted to 200 μg/mL using the stock lysate (1x).

A NanoSensor biochip (Lumera Corporation) was used for calibrating the performance of the chip. The thickness of the Au layer on the chip surface was determined to be 47.5 nm ± 0.5 nm. Cross-linking of the optical polymer layer was conducted with a <0.5% batch difference in chip binding. The optimal resonance angle was automatically adjusted using a Screen LB 991 biochip analyzer (Berthold) for the measurement of the chip.

The SPR assay is a sensitive and efficient method for identifying target proteins in complex biological samples. The use of CD117+ cell lysate as the mobile phase facilitated the detection of specific proteins of interest, whereas the CF molecule immobilized on the chip surface provided a highly specific binding site for these proteins. The combination of the SPR assay and HPLC-MS analysis were performed to identify specific protein species, which provided valuable information for further developing therapeutic interventions.

### Surface plasmon resonance (SPR) assay

The CF samples, along with four other protein samples (MGST2 and potential binding peptides), refrigerated at −80°C, were thawed under ambient temperature. Then, DMSO was added for diluting CF, which was used as a fixed-phase printing working fluid. Next, the working fluid was printed onto the 3D optical cross-linking chip using an AD1520 chip array printer (Biodot™). Four duplicate points were set for every sample, and four positive points were set for the control (Rapamycin). The printed chip was dried in a vacuum before the optical cross-linking reaction was conducted in the cross-linking machine. Then, DMF, H_2_O, and C_2_H_5_OH were shaken for 15 min and blow-dried under a stream of nitrogen. Finally, the flow cell cover was assembled for backup.

To perform protein dialysis, a commercially available protein solution was placed in a dialysis bag, and the detergent was removed with PBS. Glycerol and other additives were added to the initial storage solution. Later, a trace concentration tube was used for evaluating the protein concentration, whereas the protein content was measured using a BCA protein quantification kit and used for subsequent analyses.

Next, PBST (pH = 7.4, 0.1% Tween20) was added to those four protein sample reserves for real-time visualization, followed by dilution of the protein samples to five gradients of 10, 40, 160, 640, and 2,560 nM. Each sample was processed by circulation and testing, and PBST acted as a flow vehicle in the whole process. The analyte was passed over the chip surface at a rate of 0.5 μL/s in the interaction test. During surface regeneration, we used Glycine-HCl (pH = 2.0) as the regeneration fluid at a flow rate of 2 μL/s.

All experiments were conducted following the Standard Operating Procedure (SOP). Different gradient concentrations of each compound sample were placed on the chip surface, ranging from low to high, at a flow rate of 0.5 μL/s, binding reaction temperature of 4°C, binding time of 600 s, and dissociation time of 360 s. We used a Glycine-HCl solution at pH 2.0 as the regeneration fluid for adjusting the pH.

### Molecular dynamics (MD) simulation

The crystalline structure of Mgst2 (PDB entry: 6SSW, MAGNSILLAAVSILSACQQSYFALQVGKARLKYKVTPPAVTGSPEFERVFRAQQNCVEFYPIFIITLWMAGWYFNQVFATCLGLVYIYGRHLYFWGYSEAAKKRITGFRLSLGILALLTLLGALGIANSFLDEYLDLNIAKKLRRQF) was used to produce the original binding complexes. The Preparation Wizard module (Schrodinger, Inc.) was used to establish and determine the protein structure in the native state. After placing the protein in an environment at pH 7.0, hydrogen atoms were introduced into the protein structure based on the isoelectric points of various amino acid residues. Later, the hydrogen bonds were regulated, atomic collisions were eliminated, and charges were added to the hybrid groups to optimize the protein structure. Finally, after removing excess water molecules, the OPLS4 force field (folding to the lowest energy steady state) was used to minimize the resultant structure. After modeling and optimizing, the protein structures were analyzed based on the Ramachandran Plot and transferred to the binding site that was identified for our proposed protein structure. The binding site prediction algorithm was used to predict the Mgst2 protein region, and 20 and 11 potential binding target regions were predicted, respectively. The above-mentioned binding sites shared a huge hydrophobic area within the target region, and H bond donor and acceptor functional groups within the groove could bind to exogenous compounds.

When analyzing the binding sites, the whole protein surface was scanned to examine additional positions in the protein suitable for binding to the compound, which was later contrasted with the binding site. Then, the spatial form of the region was evaluated using the grid generation module that contained the virtual grid, and the next docking algorithm was used for grid identification and reading. For ligand docking, the super-precision simulation docking module (XP module, Schrodinger, Inc.) was used for docking segmentation. This algorithm was used to assess the binding site structure and analyze the neighboring electron cloud density and non-covalent bond acceptors and donors. While preparing compound structures based on the aforementioned processes, different compound conformations were adjusted to produce random poses according to the functional groups in every pose. Next, the level of overlap, non-covalent bond generation, and electronic cloud fusion state were selected for retaining the suitable structure, which could exist but was different from the most stable structure. Finally, the above structures were analyzed and sorted according to free energy. The stable state output was selected according to its lowest energy.

### AAV transductions and transplantation experiments

BM cells were cultured either in IMDM with GlutaMAX containing 20% fetal bovine serum, 100 IU/mL penicillin, 100 μg/mL streptomycin, 50 μM 2-mercaptoethanol, 10 ng/mL recombinant mouse interleukin (IL)-3, 25 ng/mL recombinant mouse IL-6, and 50 ng/mL recombinant mouse stem cell factor (SCF) (PeproTech, United States of America). Two hours after transduction, cells were switched to expansion mediumand grown at a density of 5 × 10^5^ cells/mL. On day 10, cells were switched to erythroid differentiation medium (IMDM, BSA, Insulin, Transferrin, Epo). BM from donor mice treated with 5-fluorouracil (FU)–treated (200 mg/kg) was transduced twice with BCR-ABL1 retrovirus by co-sedimentation in the presence of IL-3, IL-6, and SCF. Recipient mice received 1100 cGy gamma irradiation (administered by 2 divided doses of 550-cGy), 5 × 10^5^ cells were transplanted into the recipient mice by tail vein injection, as described previously (Yin et al., 2020). After transduction, mice were divided with two group and treated with xylene, the HSPCs were analyzed by flow cytometry.

### Statistical analysis

The data were presented as the arithmetic mean ± standard deviation (SD). The SPSS 26.0 software (Chicago, IL, United States of America) was used for data analysis. Repeated measurement data were initially evaluated by the repeated analysis of variance (ANOVA) when they followed a normal distribution and homogeneity. All differences were considered to be statistically significant at *p* < 0.05. The GraphPad Prism 9.0 software (La Jolla, United States of America) was used for plotting graphs.

## Results

### Coniferyl ferulate prevented the xylene-induced toxic effects on the mouse hematological system

**FIGURE 1:**
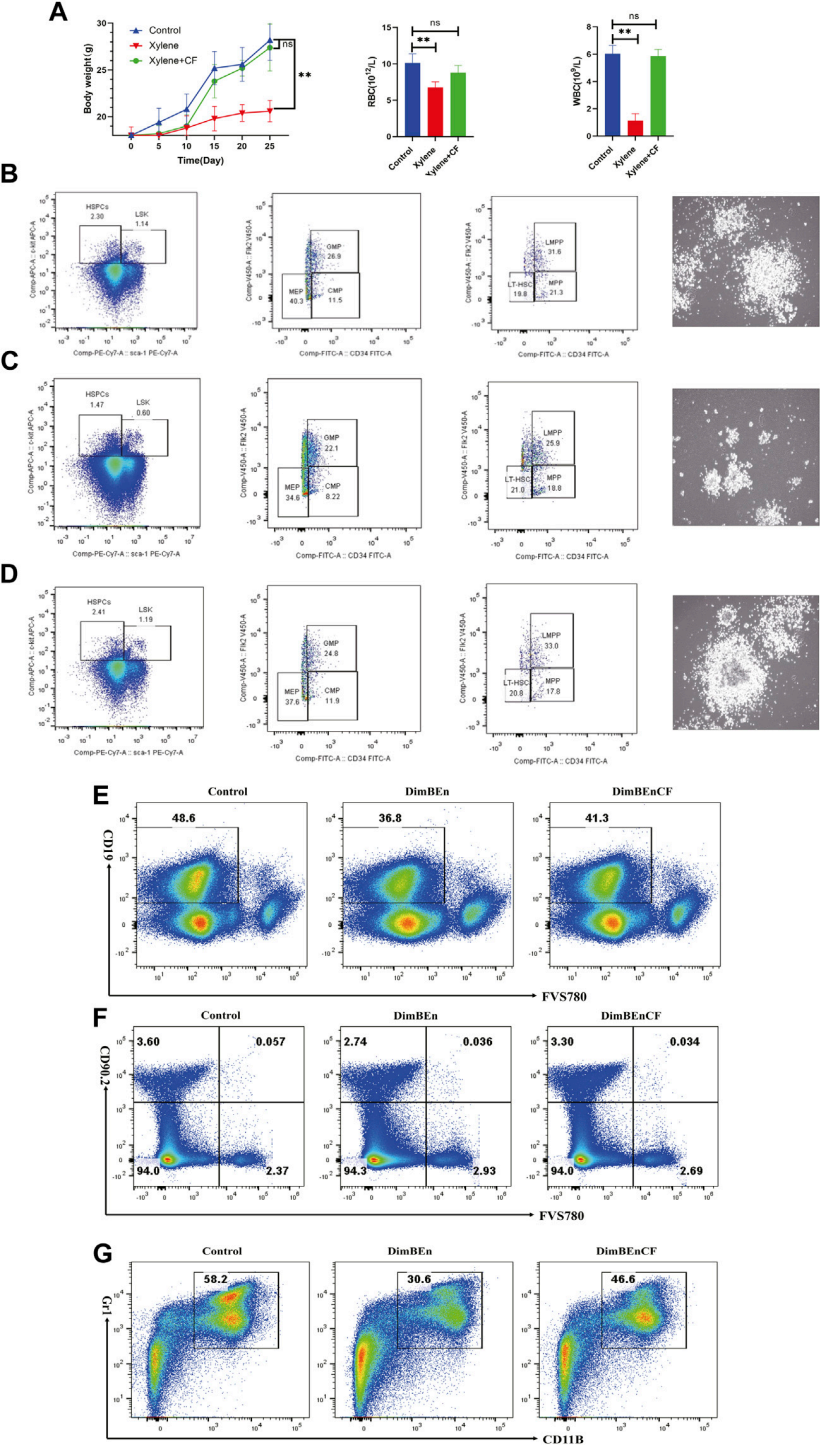
The toxic effects of xylene on the body weight, blood parameters, and percentage of hematopoietic stem and progenitor cells in mice. **(A)** The body weight of mice on the 5^th^, 10^th^, 15^th^, 20^th^, 25^th^ day of xylene exposure (left), Effects of xylene and CF+ xylene on RBC (middle) and WBC (right), WBC: white blood cell; RBC: red blood cell; The data were presented as the mean ± SD. Xylene regulates murine HSC self-renewal and differentiation can be restored by CF. Flow cytometric analysis of LSK (Lin-Sca1+c-kit+), LT-HSC (LinSca1+c‐kit+CD34-Flk2-), MPP (LinSca1+c‐kit+CD34+Flk2+), myeloid progenitors including CMP (Lin-Sca1-c-kit+CD34+CD16/32+low), granulocyte-macrophage progenitors (GMP) (Lin-Sca1-c-kit+CD34+ CD16/32+high), and MEP (Lin-Sca1-c-kit+ Sca-CD34-CD16/32-) in the BM and from Control (**B** left), Xylene (**C** left) and Xylene+CF (**D** left) group. HSPCs were plated in semisolid methylcellulose agar and colonies were counted after 14 days, Scale bars, 100 μm. Cell colony formation results shown that Xylene induced HSPCs toxicity couled be restored by CF (Control (**B** right), Xylene (**C** right) and Xylene+CF (**D** right) group. BM cells were collected from Control (**B** left), Xylene and Xylene + CFmice and stained with antibodies against Mac1, Gr1, CD19, or Thy1.2 analyzed by FACS, the damage to B cells **(E)**, T cells **(F)**, and myeloid cells **(G)** caused by xylene can be restored by CF. Student’s t-test was conducted for statistically analyzing the data; All differences were considered to be statistically significant compared to the control at P < 0.05; ** P < 0.01.

To determine whether CF could inhibit xylene-induced toxicity in the hematological system of C57BL/6 mice, the mice were pretreated with CF for 10 days before they were exposed to xylene for 15 days along with CF. The results showed that the weight of the mice decreased significantly after 15 days following the administration of 150 mg/kg b. w. Xylene. The mice that were treated with CF showed significant inhibition in the xylene-induced loss of weight (Figure 1A left). Additionally, treatment with CF inhibited the xylene-induced decrease in the level of WBC and RBC, as shown in Figure 1A (middle and right).

### Integrated analysis of the self-renewal and differentiation of mouse HSPCs

We determined the effects of CF in alleviating the toxic effects of xylene in HSCs along with progenitor compartments in mice through flow cytometry assays. Compared with Control (Figure 1B left), the proportion of LSKs in BM cells decreased considerably after 15 days following the administration of 150 mg/kg b. w. Xylene. Exposure to xylene significantly decreased the HSC-enriched LSK compartment (1.14% vs. 0.6%) along with its subsets, including MPPs (Lin-Sca1+c-kit+CD34+Flk2-) (21.3% vs. 18.8%) and LMPPs (Lin-Sca1+c-kit+CD34+Flk2+) (31.6% vs. 25.9%) in the BM (Figure 1C left). The decrease in HSC proportion induced by xylene was restored by CF, the HSC-enriched LSK compartment (0.6% vs. 1.19%) along with its subsets, including LMPPs (Lin-Sca1+c-kit+CD34+Flk2+) (25.9% vs. 33%) in the BM. Flow cytometric analysis also revealed a marked contraction of Granulocyte-Monocyte Progenitor (GMP) (Lin-Sca1+c-kit+CD34^+^ CD16/32+) (26.9% vs. 22.1%), Common Myeloid Progenitorand (CMP) (11.5% vs. 8.22%) and Multipotent Progenitor (MEP) (40.3% vs. 34.6%) in BM. In CF + xylene group, the proportion of Granulocyte-Monocyte Progenitor (GMP) (Lin-Sca1+c-kit+CD34^+^ CD16/32+) (22.1% vs. 24.8%), Common Myeloid Progenitorand (CMP) (8.22% vs. 11.9%) and Multipotent Progenitor (MEP) (34.6% vs. 37.8%) in BM were restored (Figure 1D left). The colony size of HSPCs also confirmed these results (Figures 1B–D right). These results indicated that the inhibition of HSPCs caused by xylene can be alleviated by CF.

### The damage to B cells, T cells, and myeloid cells caused by xylene can be restored by CF

We also analyzed whether CF could prevent the xylene-induced decrease in the production of mature erythroid and myeloid cells. Many mice in the xylene group showed a decrease in the proportion of myeloid cells (Gr-1+/Mac-1+) (30.6% vs. 58.2%), B cells (CD19^+^) (36.8% vs. 48.6%) and T cells (CD90.2+) (3.6% vs. 2.74%) compared to the mice that were not exposed to xylene. However, in the CF + xylene group, the population of mature myeloid (30.6% vs. 46.6%), B cells (CD19^+^) (36.8% vs. 41.3%) and T cells (CD90.2+) (2.74% vs. 3.3%) was restored to normal levels (Figures 1E–G). These results suggested that CF can reverse the xylene-induced decrease in mature myeloid, B cells and T cells *in vivo*.

### Single-cell transcriptomic profiling of BM HSPCs in mice

To determine the effects of CF on the components of HSPCs and the corresponding supporting cells in the bone marrow (BM) of mice at the transcriptome level before and after xylene induction, we submitted samples to 10xGenomics for droplet-based single-cell RNA sequencing (scRNA-seq).

**FIGURE 2:**
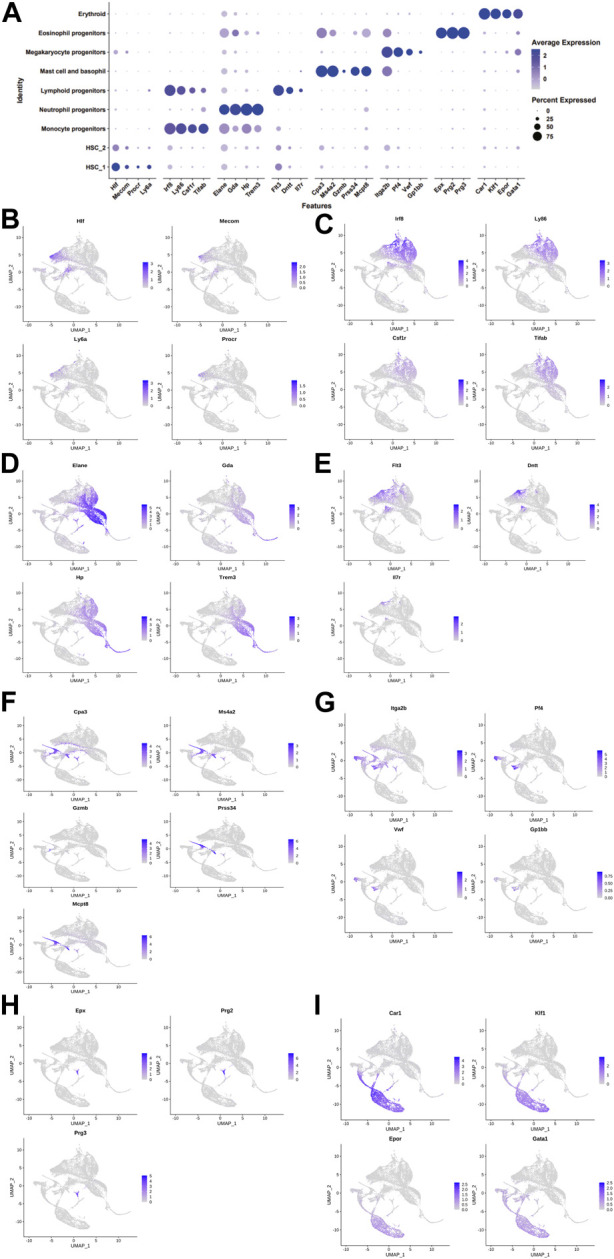
The landscape of cell clusters in HSPC samples of the bone marrow of mice was determined by the scRNA-seq analysis. **(A)**. The dot plot shows the level of expression of canonical markers in each cell cluster. UMAP visualization of eight major cell populations showing the expression of representative well-known cell-type-specific marker genes. **(B)**. Hematopoietic stem cell 1 expressing Hif, Mecom, Procr, and Ly6a. **(C)**. Monocyte progenitors expressing Lrf8, Ly86, Csf1r, and Tifab. **(D)**. Neutrophil progenitors expressing Elan, Gda, Hp, and Trem3. **(E)**. Lymphoid progenitors expressing Flt3, Dntt, and Ll7r. **(F)**. Mast cells and basophil cells expressing Ms4a2, Gzmb, Prss34, Mcpt8, and Car1. **(G)**. Megakaryocyte progenitors expressing Itga2b, Pf4, Vwf, and Gp1bb. **(H)**. Lymphoid progenitors expressing Exp, Prg2, and Prg3. **(I)**. Erythroid expressing Cpr1, Klf1, Epor, and Gata1. The differences in the proportion of cells between groups were determined by the Wilcoxon test.

We collected 6,137, 6,001, and 6840 cells from the control, DimBEn, and DimBEnCF groups, respectively. Following batch correction, cellular heterogeneity was analyzed based on uniform manifold approximation and projection (UMAP) (Figure 2A). The cells were pooled and classified into eight main clusters, including hematopoietic stem cell 1 (expressing Hif, Mecom, Procr, and Ly6a; Figure 2B), monocyte progenitors (expressing Lrf8, Ly86, Csf1r, and Tifab; Figure 2C), neutrophil progenitors (expressing Elan, Gda, Hp, and Trem3; Figure 2D), lymphoid progenitors (expressing Flt3, Dntt, and Ll7r; Figure 2E), mast cells and basophil cells (expressing Ms4a2, Gzmb, Prss34, Mcpt8, and Car1; Figure 2F), megakaryocyte progenitors (expressing Itga2b, Pf4, Vwf, and Gp1bb; Figure 2G), lymphoid progenitors (expressing Exp, Prg2, and Prg3; Figure 2H), and erythroid (expressing Cpr1, Klf1, Epor, and Gata1; Figure 2I). These results showed that the transcriptomes of HSPCs provided new ways to investigate the transcriptional landscape during the early differentiation of HSPCs at the resolution of a single cell.

### The self-renewal procedure of HSPCs was evaluated by RNA velocity analysis

As clustering does not provide any information on inter-cluster connectivity, we determined trajectories across the annotated clusters with PAGA. The arrows in the RNA velocity plot indicated the inferred developmental paths of the cells. The results indicated that the HSPCs developed into various cell types in the samples of the control group (Supplementary Figures S1A, B). In the DimBEn group, the surrounding cell types of HSPCs, especially monocyte and neutrophil progenitors, differentiated into HSPCs (Supplementary Figures S1C, D). These findings suggested that the proportion of monocyte and neutrophil progenitors probably decreased. After treatment with CF, the HSPCs differentiated into lymphoid progenitor cells (Supplementary Figures S1E, F).

### Sub-clusters were associated with the toxicity of xylene in the BM

**FIGURE 3:**
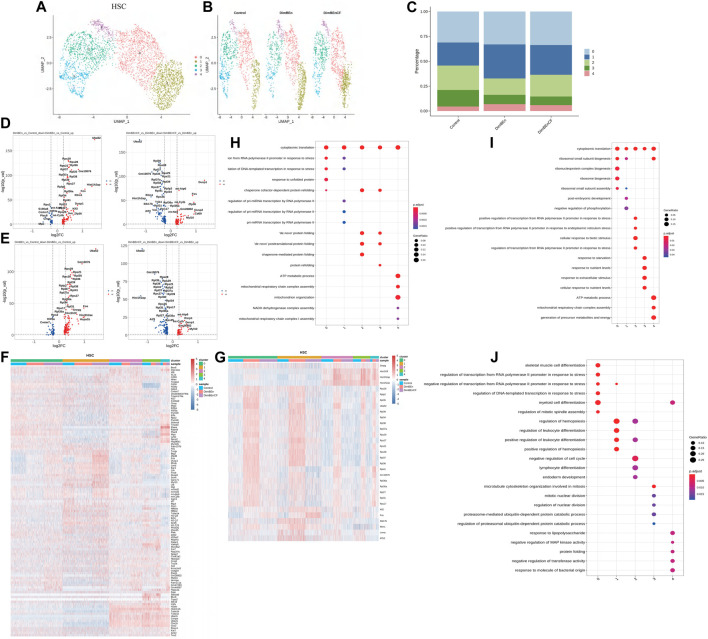
Single-cell transcriptomes of heterogeneity in hematopoietic stem cells **(A)**. The uniform manifold approximation and projection (UMAP) clustering map of mouse BM stem cell/progenitor (c-kit+). **(B)**. The UMAP plot shows the distribution of the five subcellular clusters. **(C)**. A bar plot of the proportion of cell subpopulation in three samples. The results showed that Clusters 0, 1, and 4 increased after xylene treatment, however, the changes were reversed by CF (n = 3); **p < 0.01. **(D)**. A volcano plot of differentially expressed genes between DinBEn and Control and between DinBEn and DinBEnCF **(E)**. A heat map illustrating differential gene expression. The upregulated differentially expressed genes of each cluster compared to Control and DimBEnCF; the top 20 genes were selected based on the smallest *p*-value in each cluster. **(F)**. The downregulated differentially expressed genes of each cluster compared to Control and DimBEnCF; the top 20 genes were selected based on the smallest *p*-value in each cluster. The results of the GO analysis showed that in DimBEn *versus* Control **(G)**, DimBEn *versus* DimBEnCF **(H)**, and DimBEnCF *versus* DimBEn **(I)**: Cluster 0 was associated with pressure, stimulation, and other pathway enhancement; Cluster 1 was associated with pressure and microRNA; Cluster 2 was associated with protein folding; Cluster 3 was associated with protein folding; Cluster 4 was associated with mitochondrial energy metabolism.

To investigate which cell subsets were related to the effects of xylene and CF, based on UMAP and t-SNE clustering, the HSCs were further classified into five subtypes (sub-clusters 0–4, Figures 3A, B). Then, we analyzed the number of cells in each subset between different groups. Clusters 0, 1, and 4 increased after treatment with xylene, however, this change was reversed by CF (Figure 3C). The volcano plot of differentially expressed genes (DEGs) for DinBEn *versus* Control, and DinBEn *versus* DinBEnCF are shown in Figure 3D, where the *y*-axis indicates the significance of differential expression, and larger -log_10_(p_val) values indicated that the differential expression was more significant. For each cluster, the DEGs that were upregulated in DinBEn compared to their expression in Control and DinBEnCF were selected. Then, the top 20 genes with the smallest *p*-values in each cluster were selected for plotting heat maps (Figures 3E, F). We found that some genes were differentially expressed in these sub-clusters, such as Bex6, Clec12a, and Lrf8. The results showed that Clusters 0, 1, and 4 responded to xylene stimulation; thus, we considered these three groups as pathogenic subgroups. The results of the GO analysis showed that in DimBEn *versus* Control (Figure 3G), DimBEn *versus* DimBEnCF (Figure 3H), and DimBEnCF *versus* DimBEn (Figure 3I), Cluster 0 was associated with pressure, stimulation, and other pathway enhancement; Cluster 1 was associated with pressure and microRNA; Cluster 2 was associated with protein folding; Cluster 3 was associated with protein folding; Cluster 4 was associated with mitochondrial energy metabolism. Based on this method, we analyzed monocyte progenitors, neutrophil progenitors, lymphoid progenitors, mast cells and basophils, megakaryocyte progenitors, eosinophil progenitors, and erythroid. By conducting the UMAP analysis, we also found that the number of cells in Cluster 2 decreased significantly after exposure to xylene and increased after treatment with CF. These findings suggested that this subgroup might be important for CF function and also for the process.

**FIGURE 4:**
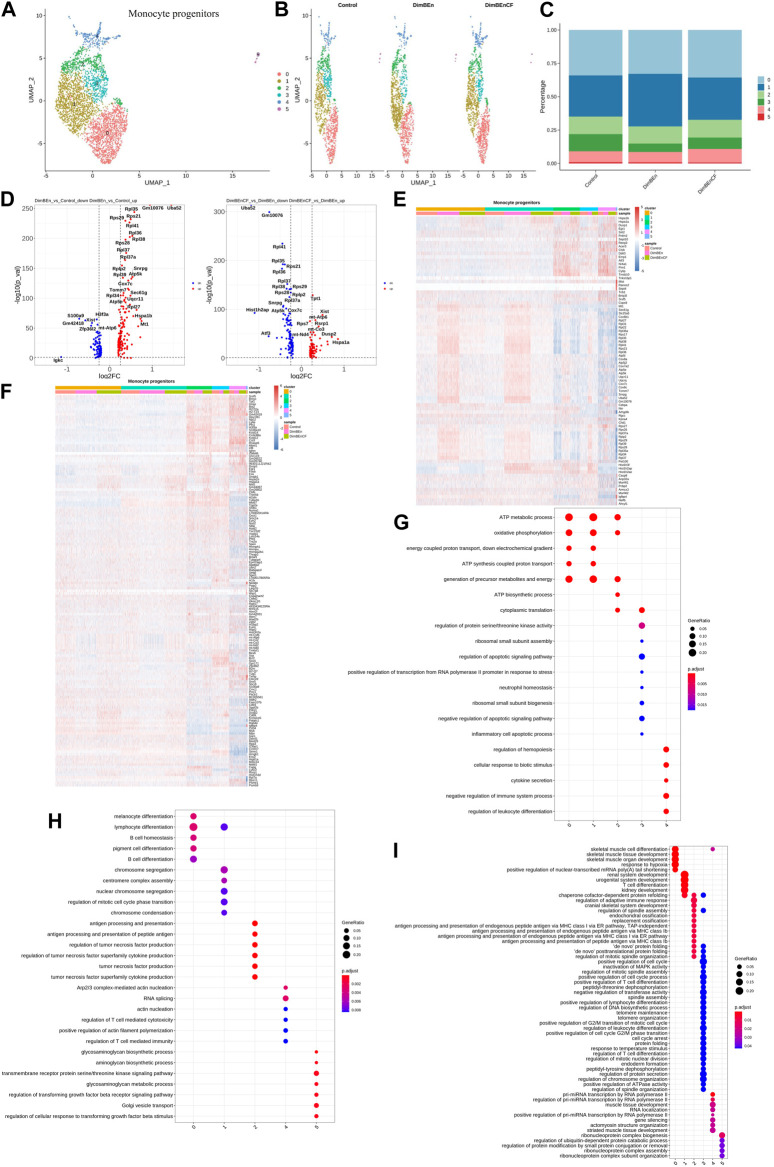
Single-cell transcriptomes of heterogeneity in monocyte progenitor cells **(A)** A uniform manifold approximation and projection (UMAP) clustering map of mouse BM monocyte progenitors from three integrated samples. **(B)**. The UMAP plot shows the distribution of the five subcellular clusters. **(C)**. A bar plot of the proportion of cell subpopulation in three samples. The results showed that Cluster 1 increased after xylene treatment, however, the changes were reversed by CF. **(D)**. A volcano plot of differentially expressed genes between DinBEn and Control, and between DinBEn and DinBEnCF **(E)**. A heat map illustrating differential gene expression. The upregulated differentially expressed genes of each cluster compared to Control and DimBEnCF; the top 20 genes were selected based on the smallest *p*-value in each cluster. **(F)**. The downregulated differentially expressed genes of each cluster compared to Control and DimBEnCF; the top 20 genes were selected based on the smallest *p*-value in each cluster. The results of the GO analysis showed that in DimBEn *versus* Control **(G)**, DimBEn *versus* DimBEnCF **(H)**, and DimBEnCF *versus* DimBEn **(I)**, Cluster 1 was related to mitochondrial energy metabolism.

Based on UMAP clustering, monocyte progenitor cells were further divided into five subtypes (sub-clusters 0–4, Figures 4A, B). Then, we analyzed the number of cells in each subset between different groups. The number of cells in Cluster 1 increased after treatment with xylene; however, this change was reversed by CF (Figure 4C). The volcano plot of DEGs for DinBEn *versus* Control and DinBEn *versus* DinBEnCF are presented in Figure 4D. For each cluster, we selected the genes that were upregulated in DinBEn compared to their expression in Control and DinBEnCF. Then, the top 20 genes with the smallest *p*-values in each cluster (such as Hspa1b, Hspa1a, and Dusp1) were selected for plotting (Figures 4E, F). These results showed that Cluster 1 can respond to xylene stimulation, and thus, we considered these groups as pathogenic subgroups. The results of the GO analysis showed that for DimBEn *versus* Control (Figure 4G), DimBEn *versus* DimBEnCF (Figure 4H), and DimBEnCF *versus* DimBEn (Figure 4I), Cluster 1 was related to mitochondrial energy metabolism. We found that Cluster 3 was significantly downregulated after exposure to xylene but upregulated after exposure to CF. We speculated that CF might exert its effects through this subgroup. The results of the GO analysis showed that this cluster was associated with pathways such as apoptosis.

**FIGURE 5:**
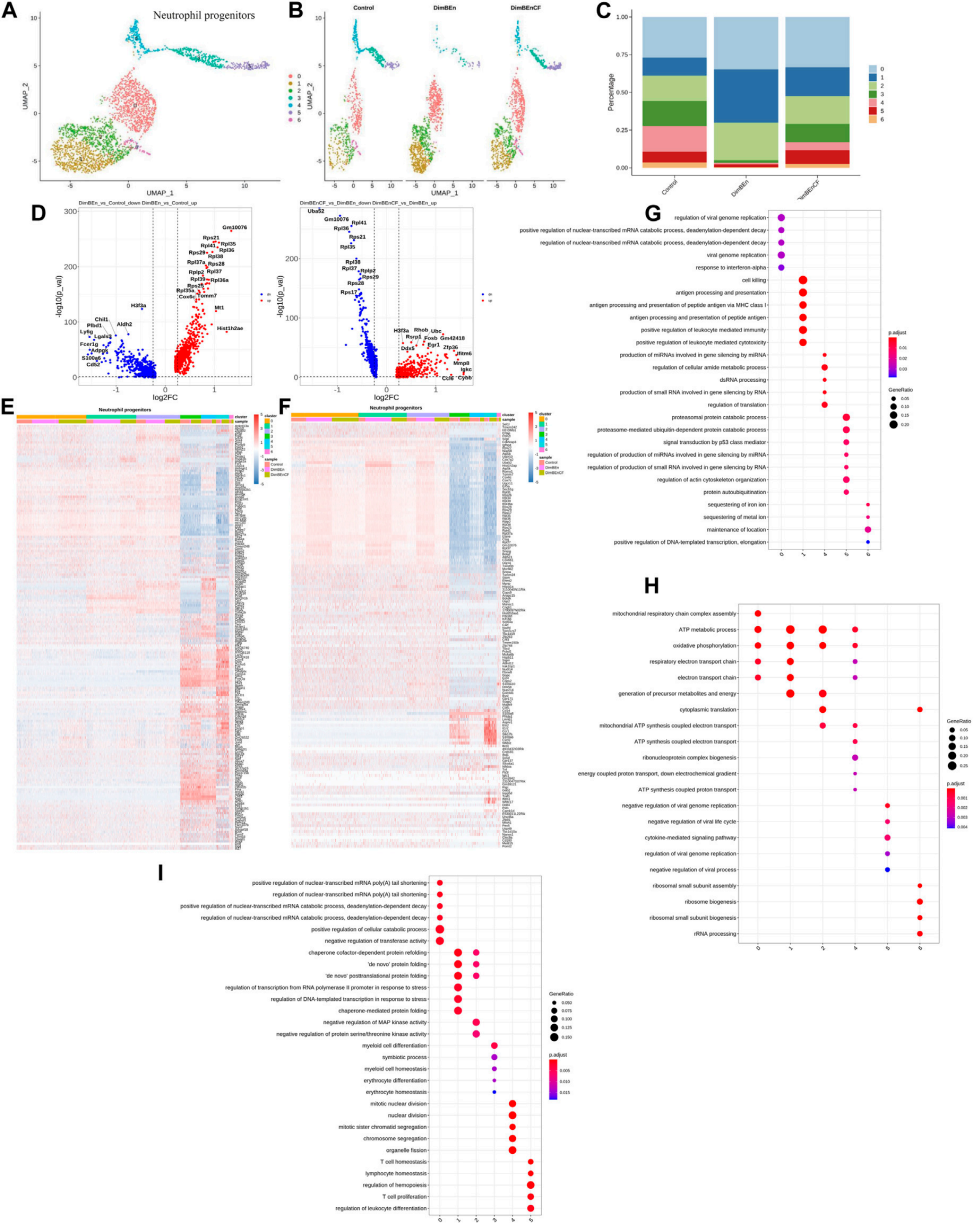
Single-cell transcriptomes of heterogeneity in neutrophil progenitor cells **(A)** A uniform manifold approximation and projection (UMAP) clustering map of mouse neutrophil progenitors cells from three integrated samples. **(B)**. The UMAP plot shows the distribution of the seven subcellular clusters. **(C)**. A bar plot of the proportion of cell subpopulation in three samples. The results showed that Clusters 0, 1, and 2 increased after xylene treatment, however, the changes were reversed by CF. **(D)**. A volcano plot of differentially expressed genes between DinBEn and Control, and between DinBEn and DinBEnCF. **(E)**. A heat map of differential gene expression. The upregulated differentially expressed genes of each cluster compared to Control and DimBEnCF; the top 20 genes were selected based on the smallest *p*-value in each cluster. **(F)**. The downregulated differentially expressed genes of each cluster compared to Control and DimBEnCF; the top 20 genes were selected based on the smallest *p*-value in each cluster. The results of the GO analysis showed that in DimBEn *versus* Control **(G)**, DimBEn *versus* DimBEnCF **(H)**, and DimBEnCF *versus* DimBEn **(I)**: Cluster 0 was associated with energy metabolism and protein folding; Cluster 1 was associated with energy metabolism and ribosome assembly; Cluster 2 was associated with energy metabolism and ribosome assembly.

According to UMAP clustering, neutrophil progenitor cells were classified into seven subtypes (sub-clusters 0–6, Figures 5A, B). Then, we analyzed the number of cells in each subset between different groups. Clusters 0, 1, and 2 increased after xylene treatment; however, the changes were reversed by CF (Figure 5C). The volcano plot of DEGs in DinBEn *versus* Control and DinBEn *versus* DinBEnCF are shown in Figure 5D. For each cluster, the DEGs that were upregulated in DinBEn compared to their expression in Control and DinBEnCF were selected. Then, the top 20 genes with the smallest *p*-values in each cluster were selected for plotting (Figures 5E, F). Then, we analyzed the number of cells in each subset between different groups and found that some genes were differentially expressed in these sub-clusters, such as Sel1i, Tmem242, and H2-Dmb1. The results showed that Clusters 0, 1, and 2 responded to xylene stimulation, and thus, we considered these three groups as pathogenic subgroups. The results of the GO analysis showed that for DimBEn *versus* Control (Figure 5G), DimBEn *versus* DimBEnCF (Figure 5H), and DimBEnCF *versus* DimBEn (Figure 5I), Cluster 0 was associated with energy metabolism and protein folding; Cluster 1 was associated with energy metabolism and ribosome assembly; cluster 2 was associated with energy metabolism and ribosome assembly. We also found that Cluster 3 was significantly inhibited by xylene, and after CF treatment, the cell percentage was restored. The results of the GO analysis showed that this cluster had a close relationship with DNA damage. Overall, the HSC Clusters 0, 1, and 4, monocyte progenitor cluster 1, and neutrophil progenitor clusters 0, 1, and 2 were considered to be the key subclusters associated with the toxic effects of xylene on HSPCs and the therapeutic effects of CF. Mitochondrial energy metabolism may contribute to the toxic effects of xylene on BM, and the regulatory effects of CF on mitochondrial energy metabolism can reverse the toxic effects on BM cells. The results of the UMAP analysis showed that other subgroups in HSPCs, including lymphoid progenitors (Supplementary Figures S2A, B, and C), mast cells and basophil cells (Supplementary Figures S2D, E, and F), megakaryocyte progenitors (Supplementary Figures S2G, H, and I), lymphoid progenitors (Supplementary Figures S2J, K, and L), and erythroid (Supplementary Figures S2M, N, and O), did not contain many xylene or CF-sensitive populations.

The GSVA was conducted for hallmark pathway enrichment for each cell, and DimBen and DimBen + CF were directly compared to the control group, separately. The results showed that the IFN-α response, DNA repair, and Pi3k/Akt signaling pathway were upregulated and the Notch, Kras, and P53 signaling pathways were downregulated in the control group of HSC1 (Supplementary Figures S3A). In HSC2, we found that Interferon-alpha response, Interferon-gamma response, and Kras signaling were upregulated and downregulated in oxidative phosphorylation, the P53 pathway, and cholesterol homeostasis in the control group (Supplementary Figure S3B). We found that apoptosis, P53, and the reactive oxygen species pathway were upregulated in the DimBen group compared to their levels in the DimBen + CF group, whereas Kras signaling and IFN-α responses were downregulated in HSC1. We also found that P53, apoptosis, and oxidative phosphorylation were upregulated in the DimBen group compared to their levels in the DimBen + CF group, whereas IFN-α responses were downregulated in HSC2. The same pattern was also found for megakaryocyte progenitors (Supplementary Figure S3C), monocyte progenitors (Supplementary Figure S3D), neutrophil progenitors (Supplementary Figure S3E), mast cells and basophils (Supplementary Figure S3F), eosinophil progenitors (Supplementary Figure S3G), and erythroid (Supplementary Figure S3H). Also, P53, IFN-α responses, Pi3k/Akt, the ROS signaling pathway, and metabolism were the most differentially expressed pathways, suggesting that these pathways of HSPCs were sensitive to xylene and CF.

### Evaluation of cell-cell interactions related to the protective effects of CF on the toxicity induced by xylene in the BM

For identifying the intercellular interactions that are conserved throughout the development of HSPCs, we constructed models that included the members participating in ligand-receptor interactions denoted as cell types found in HSPCs. We conducted permutation testing on randomized network connections, where the weighted edges were based on the fold change in the expression of receptors and ligands among the source and target population. Neutrophil progenitor cells had the most outbound and inbound connections. The results of the intercellular communication showed that the Dimben group showed significantly higher signaling networks, such as enhanced interactions of neutrophil progenitor C6 cells with additional HSPCs (e.g., HSC3) and eosinophil progenitors, via Ctsg-F2r, Anxa1-Fpr2, and Lgals9-Cd45/Cd44 ligand-receptor-based interactions (Supplementary Figures S4A, B). Some signaling pathways were significantly decreased in the Dimben group, such as decreased interactions of neutrophil progenitor cells with additional HSPCs (e.g., eosinophil progenitors), via Ccl9-Ccr1 and Ctsg-F2r ligand-receptor-based interactions (Supplementary Figures S4C, D).

### Mgst2 was the direct target of CF

**FIGURE 6:**
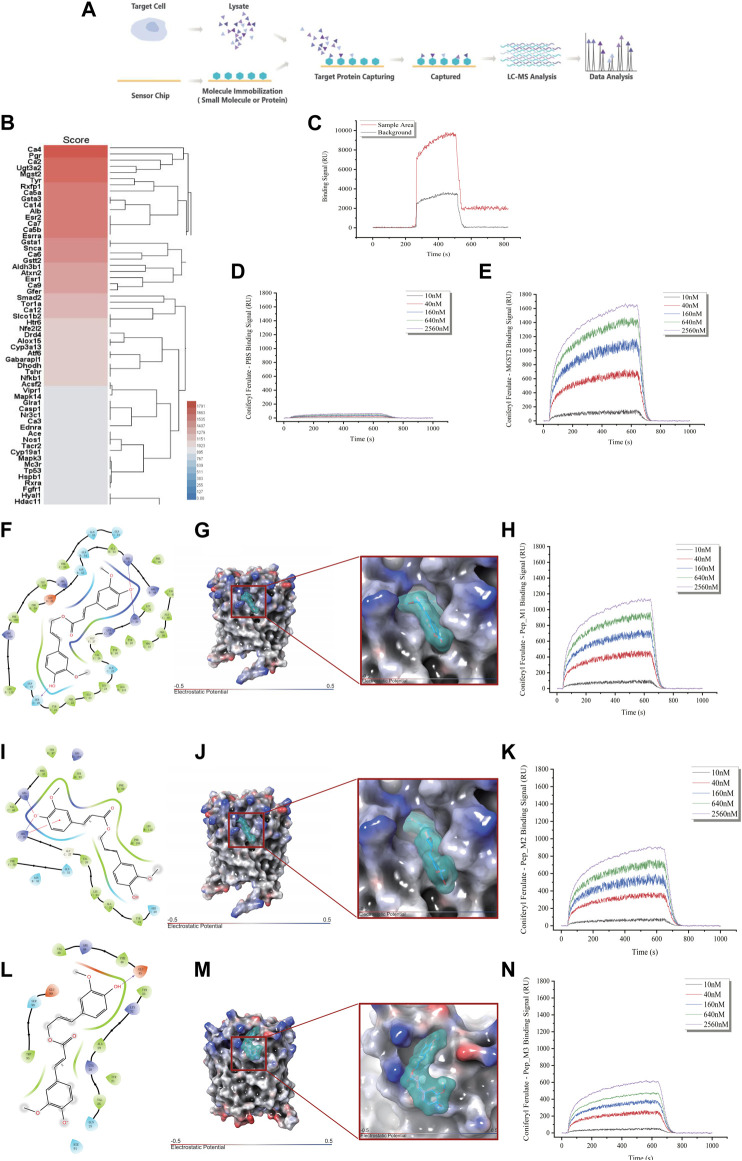
Identification of Mgst2 as the direct target of CF. **(A)**. Target identification workflow using SPR-LC-MS. **(B)**. The results of SPR-HPLC-MS showed the candidate targets that bound to CF and Mgst2. **(C)**. The results of the SPR showed the binding is specific. **(D)**. The negative control of the SPR array. **(E)**. An SPR assay was conducted to determine the binding affinity of CF to the Mgst2 protein. **(F)**. A 2D image confirming the interaction of CF with several amino acids, including QQSYFALQVGKARLKYKVTPPAVTGSPEFERVFRAQQ. **(G)**. A 3D image showing the binding of CF to the binding pocket 1. **(H)**. An SPR assay was conducted to determine the binding affinity of CF to Mgst2 peptide 1. **(I)**. A 2D image confirming the interaction of CF with several amino acids, including GKARLKYKVTPPAVTGSPEFERVFRAQQ. **(J)**. A 3D image showing the binding of CF to binding pocket 2. **(K)**. An SPR assay was conducted to determine the binding affinity of CF to Mgst2 peptide 3. **(L)** A 2D image confirming the interaction of CF with several amino acids, including AVTGSPEFERVF. **(M)** A 3D image showing the binding of CF to binding pocket **(N)**. An SPR assay was conducted to determine the binding affinity of CF to Mgst2 peptide 3.

To elucidate the mechanism behind the protective effect of CF against the toxic effects of xylene, we identified the molecular target of CF. For this, we used a chemical proteomics strategy called SPR-HPLC-MS, which helped in the direct identification of intracellular molecular targets. To confirm the specificity of the binding interactions, we performed a competition assay. We added excess unlabeled CF to the CD117+ BM cell lysate before passing it over the CF-coated chip. This led to a decrease in the SPR signal, which indicated that the unlabeled CF competed with the labeled CF for binding to the target proteins. After protein purification and identification by LC-MS/MS (Figure 6A), we found that Mgst2 was one of the targets of CF (Figure 6B). The results of the SPR (Figures 6C–E) experiments showed that Mgst2 was the direct target of CF in CD117+ BM cells. Therefore, we proposed that Mgst2 might be a promising target for alleviating xylene-induced toxicity.

### Identifying direct CF binding amino acid sites in Mgst2 proteins

For determining the CF binding sites in the Mgst2 protein, we used the Schrödinger software for MD analysis. First, we conducted structural optimization (Supplementary Figure S5A), followed by scanning of the protein-binding sites (Supplementary Figure S5B). Next, three potential CF-binding sites were detected in the Mgst2 protein. The mean docking free energy was −8.0861 kcal/mol (QQSYFALQVGKARLKYKVTPPAVTGSPEFERVFRAQQ, coring binding site: SRR) (Figures 6F–H and Supplementary Figure S5C), −7.2398 kcal/mol (GKARLKYKVTPPAVTGSPEFERVFRAQQ) (Figures 6I, J, and K and Supplementary Figure S5D), and −6.0147 (AVTGSPEFERVF) (Figures 6L–N and Supplementary Figure S5E). The root-mean-square deviation (RMSD) analysis showed the non-covalent bonded binding force (Supplementary Figures S5F, G, and H). After peptide synthesis, we examined the affinity of CF via SPR based on four proteins. Our findings indicated that peptide 1 in the Mgst2 protein interacted closely with CF. Additionally, the middle and weak interacting molecules were peptides 2 and 3, respectively. We compared the affinity between peptides and proteins and between epitope polypeptides and found that the binding site corresponded to peptide 1, and SRR was recognized as the CF-binding epitope on the Mgst2 protein, this is a non-covalent combination.

### Coniferyl ferulate (CF) alleviated the toxic effects of xylene on BM by inhibiting the expression of Mgst2

**FIGURE 7:**
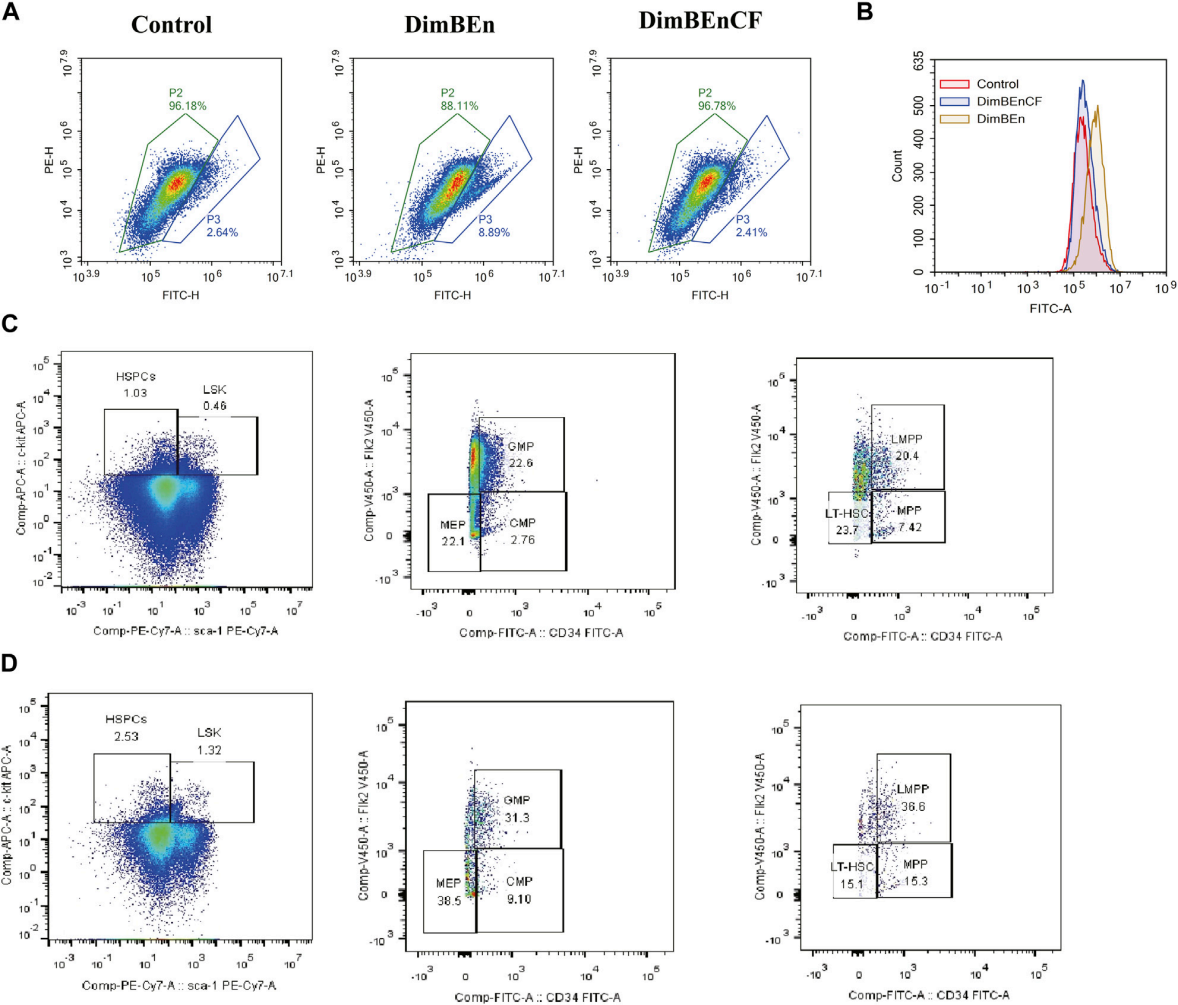
Coniferyl ferulate (CF) protected the BM from the toxic effects of xylene by inhibiting the expression of Mgst2 **(A)**. CF reversed the xylene-induced decrease in mitochondrial transmembrane potential **(B)**. CF reversed the xylene-induced decrease in ROS production. Xylene regulates murine HSC self-renewal and differentiation can be restored by inhibiting Mgst2. Flow cytometric analysis of LSK (Lin-Sca1+c-kit+), LT-HSC (Lin-Sca1+c‐kit+CD34-Flk2-), and MPP (Lin-Sca1+c‐kit+CD34+Flk2+) in the BM from controls, flow cytometric analysis of myeloid progenitors including CMP (Lin-Sca1-c-kit+CD34^+^CD16/32+low), granulocyte-macrophage progenitors (GMP) (Lin-Sca1-c-kit+CD34^+^ CD16/32+high), and MEP (Lin-Sca1-c-kit + Sca-CD34^−^CD16/32-) in the BM and from Xylene **(C)** and Xylene + Mgst2 down expression **(D)** group.

We analyzed the expression of Mgst2 in HSPCs (Supplementary Figure 6A) and found that Mgst2 was expressed in HSCs (Supplementary Figure 6B), monocyte progenitors (Supplementary Figure 6C), and neutrophil progenitors. They were especially overexpressed in neutrophil progenitors (Supplementary Figure 6D). However, they were rarely expressed in lymphoid progenitors (Supplementary Figure 6E), mast cells and basophils (Supplementary Figure 6F), megakaryocyte progenitors (Supplementary Figure 6G), and eosinophil progenitors (Supplementary Figure 6H). Based on these results, we speculated that HSCs, monocyte progenitors, and neutrophil progenitors might be the key cell populations influenced by CF. Additionally, CF reversed the xylene-induced decrease in mitochondrial transmembrane potential (Figure 7A) and ROS production (Figure 7B). Finally, the results of this study showed that the inhibition of Mgst2 reversed the toxic effects of xylene on the BM in mice. We generated Mgst2 knockdown mice using AAV6 and found a significant reduction in xylene-induced hematotoxicity in bone marrow-transplanted mice with Mgst2 knockdown. The decrease in HSC proportion induced by xylene was restored by inhibiting Mgst2 expression in BM, the HSC-enriched LSK compartment (0.46% vs. 1.32%) along with its subsets, including LMPPs (Lin-Sca1+c-kit+CD34+Flk2+) (20.4% vs. 36.6%) in the BM. Flow cytometric analysis also revealed a marked contraction of Granulocyte-Monocyte Progenitor (GMP) (Lin-Sca1+c-kit+CD34^+^ CD16/32+) (22.6% vs. 31.3%), Common Myeloid Progenitorand (CMP) (11.5% vs. 8.22%) and Multipotent Progenitor (MEP) (38.5% vs. 22.1%) in BM (Figures 7C, D). These findings indicated that CF is a potential therapeutic agent that can alleviate the toxic effects of xylene on the BM by Mgst2.

## Discussion

Xylene-induced injury to HSPCs is an important event in hematopoietic toxicity (Partha et al., 2022). Xylene can decrease the number and colony formation capacity of HSPCs and promote their apoptosis, alterations in the cell cycle, and damage to the DNA (Liao et al., 2022; Eom et al., 2023). CF is a phenolic acid compound that is extensively distributed in Angelica sinensis, which has various pharmacological effects (Hao et al., 2021). However, studies on its role in treating xylene-induced hematopoietic toxicity are rare. In this study, we analyzed the role of CF in alleviating the hematopoietic toxicity induced by xylene using an animal model. We also analyzed its associated mechanisms via single-cell transcriptome sequencing. We found that CF bound to Mgst2 protein and inhibited mitochondrial energy metabolism. The mechanism by which CF alleviated xylene-induced hematopoietic toxicity was novel.

Before transcriptome sequencing, conventional toxicology assays were conducted to evaluate xylene-induced hematotoxicity. The considerable reduction in the content of peripheral blood WBCs and RBCs in the xylene group was similar to findings of another study (Sun et al., 2020). We also found that after CF treatment, the decrease in the content of WBC and RBC caused by xylene was restored. These findings indicated that CF can protect bone marrow cells from the toxic effects of xylene and, thus, help maintain normal blood cell counts. The results of the laboratory and epidemiological studies showed that exposure to benzene influences the BM, which can cause multiple hematopoietic anomalies, including a decrease in the peripheral blood lymphocyte/neutrophil/platelet counts, multiple myeloma, acute myeloid leukemia, and aplastic anemia (Scharf et al., 2020). Our findings were similar to those reported in previous studies, which also found that xylene can decrease the number of lymphocyte and neutrophil progenitor cells in the bone marrow, and this toxic effect can be inhibited by CF. These findings further indicated that CF is a promising therapeutic agent for treating bone marrow damage caused by xylene.

Workers have a high risk of exposure to benzene in the occupational setting, and long-term exposure to benzene can impair blood and immune cell count (Durmusoglu et al., 2010; Bahadar et al., 2014). As the whole transcriptome can be analyzed using data-driven methods, single-cell genomics might be used to transform the ability to define the cellular state and cell type (Liu et al., 2023; Zhou et al., 2023). HSPCs are an extremely heterogeneous group (Zhou et al., 2020). Previous studies have not reported which subgroups can respond to the toxic effects of xylene. Moreover, no study has investigated which subgroups respond after drug treatment. In this study, we subdivided the major classes of HPSCs into smaller subgroups and identified some pathogenic subgroups. These findings provided new insights into the mechanisms by which specific subgroups respond to the toxic effects of xylene. This information is valuable for investigating benzene-induced hematotoxicity.

Neutrophils are the most abundant in circulation, and they are also the initial responders at the site of the infection, where they swallow pathogenic microorganisms through degranulation, phagocytosis, and the production of neutrophil extracellular traps (Hirschhorn et al., 2023; Roy et al., 2023). Neutrophils in vertebrates follow a conservative development process. They can be obtained from neutrophil progenitors, which differentiate from myeloblasts into mature neutrophils (Wigerblad and Kaplan, 2023). Toxic effects such as the deficiency of neutrophils or leukopenia experienced during chemotherapy are associated with favorable clinical outcomes in several types of cancer (Gurney, 2002). In this study, we found that xylene induced a decrease in the number of neutrophils, and coniferyl ferulate reversed this change. The toxic effects of xylene might lead to the inhibition of the differentiation of hematopoietic stem cells into neutrophil progenitor cells. The deficiency of neutrophils can decrease resistance to microbes, such as bacteria, fungi, and viruses, which can increase the risk of developing infectious diseases, such as pneumonia, meningitis, and sepsis. Neutrophil deficiency might also result in symptoms such as anemia, bleeding, and fever.

Monocytes strongly influence adaptive and innate immunity in circulation, and they are required for inflammation, tissue remodeling, and immune defense (Friedmann-Morvinski and Hambardzumyan, 2023). They are innate immune cells that develop in the BM and can be persistently released in circulation. They enter tissues after inflammatory cues are released or changes in homeostasis occur (Robinson et al., 2021). Monocytes exhibit high plasticity and may differentiate into various monocyte-derived cells in tissues for replacing resident tissue macrophages, enhancing inflammation, or promoting inflammatory responses (Richoz et al., 2022). They strongly influence tissue homeostasis and pathogenic or productive immunity (Guilliams et al., 2018). For maintaining homeostasis, monocytes can migrate to tissues and differentiate into specialized monocytes or macrophages according to the environmental signals. Also, they may accumulate and contribute to the monocyte reservoir (Shi and Pamer, 2011). During inflammation and infection, they enter the affected tissue quickly. Some studies have investigated the fates of monocytes with special functions under different inflammatory settings (Mulder et al., 2021). Monocytes are a part of the immune system and can identify and eliminate pathogens. A lack of monocytes weakens the immune system and affects its function, making the body more susceptible to infections. It may also cause feelings of fatigue and weakness because monocytes participate in regulating energy metabolism and maintaining overall health. They also participate in regulating the inflammatory response. Monocyte deficiency may lead to uncontrolled inflammatory responses, causing inflammation. It may also increase the risk of development of other diseases, such as cardiovascular disease and cancer.

To summarize, a lack of monocytes can seriously affect overall health, and timely diagnosis and treatment are necessary. In this study, we identified key pathogenic subpopulations of monocyte progenitor cells that respond to the toxic effects of xylene, providing new insights into the treatment of xylene-induced hematotoxicity.

Drug target identification is a key method for determining the mechanism of action of drugs (Yin et al., 2020). In this study, using the SPR-LC/MS method, we identified the key target protein of CF that mediates its effects against the hematotoxic effects of xylene. Based on the findings of other studies and our single-cell transcriptome sequencing results, we found that xylene-induced hematotoxicity is related to ROS and mitochondrial metabolism (Huang et al., 2021; Zhang et al., 2022). Therefore, we analyzed proteins associated with ROS and mitochondrial metabolism among the target proteins. Mgst2 is an enzyme that is responsible for catalyzing certain glutathione (GSH)-mediated reactions (Thulasingam et al., 2021). For example, Mgst2 binds to GSH to generate thiolate for peroxide reduction or combines with xenobiotic electrophiles, like 1-chloro-2, 4-dinitrobenzene (CDNB), or with endogenous epoxide leukotriene A4 (LTA4) to produce LTC4, a smooth muscle contractile agent (Rubinstein and Dvash, 2018). Mgst2 is different from its closest relative LTC4S, and it can effectively catalyze CDNB-conjugation, which is commonly observed in enzymes related to detoxification reactions, including soluble glutathione transferases or Mgst1. LTC4S and Mgst2 are membrane-associated proteins that belong to the eicosanoid and glutathione (MAPEG) metabolic protein family. Mgst2 participates in the transcellular biosynthesis of LTC4 and the catalysis of GSH-mediated reduction reaction depending on peroxidase activity (Dvash et al., 2015). Its GSH-mediated peroxidase activity leads to oxidative modification by reducing fatty acid hydroperoxide and phospholipid. ROS are potent antimicrobial agents and can cause inflammation during several biological processes (Wang et al., 2021). Mgst2 can mitigate ROS-mediated lipid peroxidation, which strongly affects the innate immunity of the sea cucumber (Kelner et al., 2014). We found that xylene promoted the production of ROS, and after CF treatment, ROS production was inhibited. Excessive ROS production can damage organs, indicating that the toxic effects of xylene on HSPCs can be induced by oxidative stress, and CF can reverse these changes. By identifying the target, we showed that Mgst2 is a target protein associated with xylene-induced cytotoxicity. The results of the single-cell transcriptome analysis showed that the expression of Mgst2 in neutrophil progenitor cells was initially high, but its level decreased after xylene treatment. Based on these findings, we hypothesized that Mgst2 strongly affects the toxic effects of xylene on HSPCs.

To summarize, in this study, we found that CF can alleviate the toxic effects of xylene *in vivo* and *in vitro.* Our findings provided new evidence, which suggested that CF protects HSPCs from the toxic effects of xylene by inhibiting mitochondrial metabolism in monocytes and neutrophil progenitor subcluster at the single-cell resolution. Based on the results of MD, SPR assay, Mgst2 was found to be the direct target of CF. Regarding the mechanism of action, we speculated that CF is directly bound to Mgst2 to inhibit mitochondrial metabolism, which alleviated the toxic effects of xylene on HSPCs. Therefore, Mgst2 might be the therapeutic target that is regulated to manage xylene-induced toxicity. Our findings might serve as the preclinical rationale for the application of CF as a therapeutic agent for alleviating the toxic effects of xylene on HSPCs.

## Data Availability

The original contributions presented in the study are included in the article/Supplementary Material, further inquiries can be directed to the corresponding authors.

## References

[B1] Bahadar H. Mostafalou S. Abdollahi M. (2014). Current understandings and perspectives on non-cancer health effects of benzene: a global concern. Toxicol. Appl. Pharmacol. 276 (2), 83–94. 10.1016/j.taap.2014.02.012 24589379

[B2] Crippa S. Conti A. Vavassori V. Ferrari S. Beretta S. Rivis S. (2023). Mesenchymal stromal cells improve the transplantation outcome of CRISPR-Cas9 gene-edited human HSPCs. J. Am. Soc. Gene Ther. 31 (1), 230–248. 10.1016/j.ymthe.2022.08.011 PMC9840125 35982622

[B3] Durmusoglu E. Taspinar F. Karademir A. (2010). Health risk assessment of BTEX emissions in the landfill environment. J. Hazard. Mater. 176 (1-3), 870–877. 10.1016/j.jhazmat.2009.11.117 20022163

[B4] Dvash E. Har-Tal M. Barak S. Meir O. Rubinstein M. (2015). Leukotriene C4 is the major trigger of stress-induced oxidative DNA damage. Nat. Commun. 6, 10112. 10.1038/ncomms10112 26656251 PMC4682057

[B5] Eom H. Kim S. Oh S. E. (2023). Evaluation of joint toxicity of BTEX mixtures using sulfur-oxidizing bacteria. J. Environ. Manag. 325 (Pt A), 116435. 10.1016/j.jenvman.2022.116435 36270122

[B6] Friedmann-Morvinski D. Hambardzumyan D. (2023). Monocyte-neutrophil entanglement in glioblastoma. J. Clin. investigation 133 (1), e163451. 10.1172/JCI163451 PMC9797336 36594465

[B7] Fujimori K. Uno S. Kuroda K. Matsumoto C. Maehara T. (2022). Leukotriene C4 synthase is a novel PPARγ target gene, and leukotriene C4 and D4 activate adipogenesis through cysteinyl LT1 receptors in adipocytes. Mol. Cell Res. 1869 (3), 119203. 10.1016/j.bbamcr.2021.119203 34968576

[B8] Gong W. Zhou Y. Gong W. Qin X. (2020). Coniferyl ferulate exerts antidepressant effect via inhibiting the activation of NMDAR-CaMKII-MAPKs and mitochondrial apoptotic pathways. J. Ethnopharmacol. 251, 112533. 10.1016/j.jep.2019.112533 31911178

[B9] Guilliams M. Mildner A. Yona S. (2018). Developmental and functional heterogeneity of monocytes. Immunity 49 (4), 595–613. 10.1016/j.immuni.2018.10.005 30332628

[B10] Gurney H. (2002). How to calculate the dose of chemotherapy. Br. J. cancer 86 (8), 1297–1302. 10.1038/sj.bjc.6600139 11953888 PMC2375356

[B11] Hao W. Z. Ma Q. Y. Tao G. Huang J. Q. Chen J. X. (2021). Oral coniferyl ferulate attenuated depression symptoms in mice via reshaping gut microbiota and microbial metabolism. Food and Funct. 12 (24), 12550–12564. 10.1039/d1fo02655k 34812830

[B12] Hassan H. A. Aly A. A. (2018). Isolation and characterization of three novel catechol 2,3-dioxygenase from three novel haloalkaliphilic BTEX-degrading Pseudomonas strains. Int. J. Biol. Macromol. 106, 1107–1114. 10.1016/j.ijbiomac.2017.08.113 28847603

[B13] Hirschhorn D. Budhu S. Kraehenbuehl L. Gigoux M. Schröder D. Chow A. (2023). T cell immunotherapies engage neutrophils to eliminate tumor antigen escape variants. Cell 186 (7), 1432–1447. 10.1016/j.cell.2023.03.007 37001503 PMC10994488

[B14] Huang H. Jiang Y. Zhao J. Li S. Schulz S. Deng L. (2021). BTEX biodegradation is linked to bacterial community assembly patterns in contaminated groundwater ecosystem. J. Hazard. Mater. 419, 126205. 10.1016/j.jhazmat.2021.126205 34216964

[B15] Huang Y. Cheng M. Wang X. Dong H. Gao J. (2022). Dang Gui Bu Xue Tang, a conventional Chinese herb decoction, ameliorates radiation-induced heart disease via Nrf2/HMGB1 pathway. Front. Pharmacol. 13, 1086206. 10.3389/fphar.2022.1086206 36699071 PMC9868149

[B16] Inesta-Vaquera F. Miyashita L. Grigg J. Henderson C. J. Wolf C. R. (2023). Defining the *in vivo* mechanism of air pollutant toxicity using murine stress response biomarkers. Sci. total Environ. 888, 164211. 10.1016/j.scitotenv.2023.164211 37196967

[B17] Kang Y. J. Tan H. Y. Lee C. Y. Cho H. (2021). An air particulate pollutant induces neuroinflammation and neurodegeneration in human brain models. Adv. Sci. (Weinh). 8 (21), e2101251. 10.1002/advs.202101251 34561961 PMC8564420

[B18] Kelner M. J. Diccianni M. B. Yu A. L. Rutherford M. R. Estes L. A. Morgenstern R. (2014). Absence of MGST1 mRNA and protein expression in human neuroblastoma cell lines and primary tissue. Free Radic. Biol. Med. 69, 167–171. 10.1016/j.freeradbiomed.2014.01.021 24486338 PMC4010302

[B19] Lan Q. Zhang L. Li G. Vermeulen R. Weinberg R. S. Dosemeci M. (2004). Hematotoxicity in workers exposed to low levels of benzene. Sci. (New York, N.Y.) 306 (5702), 1774–1776. 10.1126/science.1102443 PMC1256034 15576619

[B20] Liao Q. Zhang Y. Ma R. Zhang Z. Ji P. Xiao M. (2022). Risk assessment and dose-effect of co-exposure to benzene, toluene, ethylbenzene, xylene, and styrene (BTEXS) on pulmonary function: a cross-sectional study. Environ. Pollut. 310, 119894. 10.1016/j.envpol.2022.119894 35932901

[B21] Liu Y. M. Ge J. Y. Chen Y. F. Liu T. Chen L. Liu C. C. (2023). Combined single-cell and spatial transcriptomics reveal the metabolic evolvement of breast cancer during early dissemination. Adv. Sci. (Weinh). 10 (6), e2205395. 10.1002/advs.202205395 36594618 PMC9951304

[B22] Mokammel A. Rostami R. Niazi S. Asgari A. Fazlzadeh M. (2022). BTEX levels in rural households: heating system, building characteristic impacts and lifetime excess cancer risk assessment. Environ. Pollut. 298, 118845. 10.1016/j.envpol.2022.118845 35031402

[B23] Mosmeri H. Gholami F. Shavandi M. Dastgheib S. M. M. Alaie E. (2019). Bioremediation of benzene-contaminated groundwater by calcium peroxide (CaO(2)) nanoparticles: continuous-flow and biodiversity studies. J. Hazard. Mater. 371, 183–190. 10.1016/j.jhazmat.2019.02.071 30851671

[B24] Mulder K. Patel A. A. Kong W. T. Piot C. Halitzki E. Dunsmore G. (2021). Cross-tissue single-cell landscape of human monocytes and macrophages in health and disease. Immunity 54 (8), 1883–1900.e5. 10.1016/j.immuni.2021.07.007 34331874

[B25] Nishida-Aoki N. Mori H. Kuroda K. Ueda M. (2015). Activation of the mitochondrial signaling pathway in response to organic solvent stress in yeast. Curr. Genet. 61 (2), 153–164. 10.1007/s00294-014-0463-9 25487302

[B26] Partha D. B. Cassidy-Bushrow A. E. Huang Y. (2022). Global preterm births attributable to BTEX (benzene, toluene, ethylbenzene, and xylene) exposure. Sci. total Environ. 838 (Pt 4), 156390. 10.1016/j.scitotenv.2022.156390 35654176

[B27] Richoz N. Tuong Z. K. Loudon K. W. Patiño-Martínez E. Ferdinand J. R. Portet A. (2022). Distinct pathogenic roles for resident and monocyte-derived macrophages in lupus nephritis. JCI insight 7 (21), e159751. 10.1172/jci.insight.159751 36345939 PMC9675473

[B28] Robinson A. Han C. Z. Glass C. K. Pollard J. W. (2021). Monocyte regulation in homeostasis and malignancy. Trends Immunol. 42 (2), 104–119. 10.1016/j.it.2020.12.001 33446416 PMC7877795

[B29] Roy S. Halder M. Ramprasad P. Dasgupta S. Singh Y. Pal D. (2023). Oxidized pullulan exhibits potent antibacterial activity against *S. aureus* by disrupting its membrane integrity. Int. J. Biol. Macromol. 249, 126049. 10.1016/j.ijbiomac.2023.126049 37517748

[B30] Rubinstein M. Dvash E. (2018). Leukotrienes and kidney diseases. Curr. Opin. Nephrol. Hypertens. 27 (1), 42–48. 10.1097/MNH.0000000000000381 29059080 PMC5732635

[B31] Salimi A. Talatappe B. S. Pourahmad J. (2017). Xylene induces oxidative stress and mitochondria damage in isolated human lymphocytes. Toxicol. Res. 33 (3), 233–238. 10.5487/TR.2017.33.3.233 28744355 PMC5523563

[B32] Scharf P. Broering M. F. Oliveira da Rocha G. H. Farsky S. H. P. (2020). Cellular and molecular mechanisms of environmental pollutants on hematopoiesis. Int. J. Mol. Sci. 21 (19), 6996. 10.3390/ijms21196996 32977499 PMC7583016

[B33] Shi C. Pamer E. G. (2011). Monocyte recruitment during infection and inflammation. Nat. Rev. Immunol. 11 (11), 762–774. 10.1038/nri3070 21984070 PMC3947780

[B34] Singh M. P. Ram K. R. Mishra M. Shrivastava M. Saxena D. K. Chowdhuri D. K. (2010). Effects of co-exposure of benzene, toluene and xylene to *Drosophila melanogaster*: alteration in hsp70, hsp60, hsp83, hsp26, ROS generation and oxidative stress markers. Chemosphere 79 (5), 577–587. 10.1016/j.chemosphere.2010.01.054 20188393

[B35] Snyder R. (2012). Leukemia and benzene. Int. J. Environ. Res. public health 9 (8), 2875–2893. 10.3390/ijerph9082875 23066403 PMC3447593

[B36] Sun R. Liu M. Xu K. Pu Y. Huang J. Liu J. (2022). Ferroptosis is involved in the benzene-induced hematotoxicity in mice via iron metabolism, oxidative stress and NRF2 signaling pathway. Chemico-biological Interact. 362, 110004. 10.1016/j.cbi.2022.110004 35661779

[B37] Sun R. Xu K. Ji S. Pu Y. Man Z. Ji J. (2020). Benzene exposure induces gut microbiota dysbiosis and metabolic disorder in mice. Sci. total Environ. 705, 135879. 10.1016/j.scitotenv.2019.135879 31972927

[B38] Thulasingam M. Orellana L. Nji E. Ahmad S. Rinaldo-Matthis A. Haeggström J. Z. (2021). Crystal structures of human MGST2 reveal synchronized conformational changes regulating catalysis. Nat. Commun. 12 (1), 1728. 10.1038/s41467-021-21924-8 33741927 PMC7979937

[B39] Vermeulen R. Lan Q. Qu Q. Linet M. S. Zhang L. Li G. (2023). Nonlinear low dose hematotoxicity of benzene; a pooled analyses of two studies among Chinese exposed workers. Environ. Int. 177, 108007. 10.1016/j.envint.2023.108007 37290291

[B40] Wang B. Xu S. Sun Q. Li X. Wang T. Xu K. (2022). Let-7e-5p, a promising novel biomarker for benzene toxicity, is involved in benzene-induced hematopoietic toxicity through targeting caspase-3 and p21. Ecotoxicol. Environ. Saf. 246, 114142. 10.1016/j.ecoenv.2022.114142 36193590

[B41] Wang P. Yang W. Guo H. Dong H. P. Guo Y. Y. Gan H. (2021). IL-36γ and IL-36Ra reciprocally regulate NSCLC progression by modulating GSH homeostasis and oxidative stress-induced cell death. Adv. Sci. (Weinh). 8 (19), e2101501. 10.1002/advs.202101501 34369094 PMC8498882

[B42] Wigerblad G. Kaplan M. J. (2023). Neutrophil extracellular traps in systemic autoimmune and autoinflammatory diseases. Nat. Rev. Immunol. 23 (5), 274–288. 10.1038/s41577-022-00787-0 36257987 PMC9579530

[B43] Xia J. Liu M. Zhu C. Liu S. Ai L. Ma D. (2023). Activation of lineage competence in hemogenic endothelium precedes the formation of hematopoietic stem cell heterogeneity. Cell Res. 33 (6), 448–463. 10.1038/s41422-023-00797-0 37016019 PMC10235423

[B44] Yin Z. Huang G. Gu C. Liu Y. Yang J. Fei J. (2020). Discovery of berberine that targetedly induces autophagic degradation of both BCR-ABL and BCR-ABL T315I through recruiting LRSAM1 for overcoming imatinib resistance. Clin. cancer Res. 26 (15), 4040–4053. 10.1158/1078-0432.CCR-19-2460 32098768

[B45] Yin Z. Su R. Ge L. Wang X. Yang J. Huang G. (2023). Single-cell resolution reveals RalA GTPase expanding hematopoietic stem cells and facilitating of BCR-ABL1-driven leukemogenesis in a CRISPR/Cas9 gene editing mouse model. Int. J. Biol. Sci. 19 (4), 1211–1227. 10.7150/ijbs.76993 36923939 PMC10008703

[B46] Zhang W. Wang J. Liu Z. Zhang L. Jing J. Han L. (2022). Iron-dependent ferroptosis participated in benzene-induced anemia of inflammation through IRP1-DHODH-ALOX12 axis. Free Radic. Biol. Med. 193 (Pt 1), 122–133. 10.1016/j.freeradbiomed.2022.10.273 36244588

[B47] Zhou T. Chen Y. Liao Z. Zhang L. Su D. Li Z. (2023). Spatiotemporal characterization of human early intervertebral disc formation at single-cell resolution. Adv. Sci. (Weinh). 10 (14), e2206296. 10.1002/advs.202206296 36965031 PMC10190614

[B48] Zhou Y. Zhu X. Dai Y. Xiong S. Wei C. Yu P. (2020). Chemical cocktail induces hematopoietic reprogramming and expands hematopoietic stem/progenitor cells. Adv. Sci. (Weinh). 7 (1), 1901785. 10.1002/advs.201901785 31921559 PMC6947705

