# Supplementary material for: Coniferyl ferulate alleviate xylene-caused hematopoietic stem and progenitor cell toxicity by Mgst2
Source: Front Pharmacol. 2024 Mar 8;15:1334445. doi: 10.3389/fphar.2024.1334445 (PMC10957570; doi:10.3389/fphar.2024.1334445)
Supplement: Supplementary file 1 [file Table1.DOCX]

**Supplemental information**


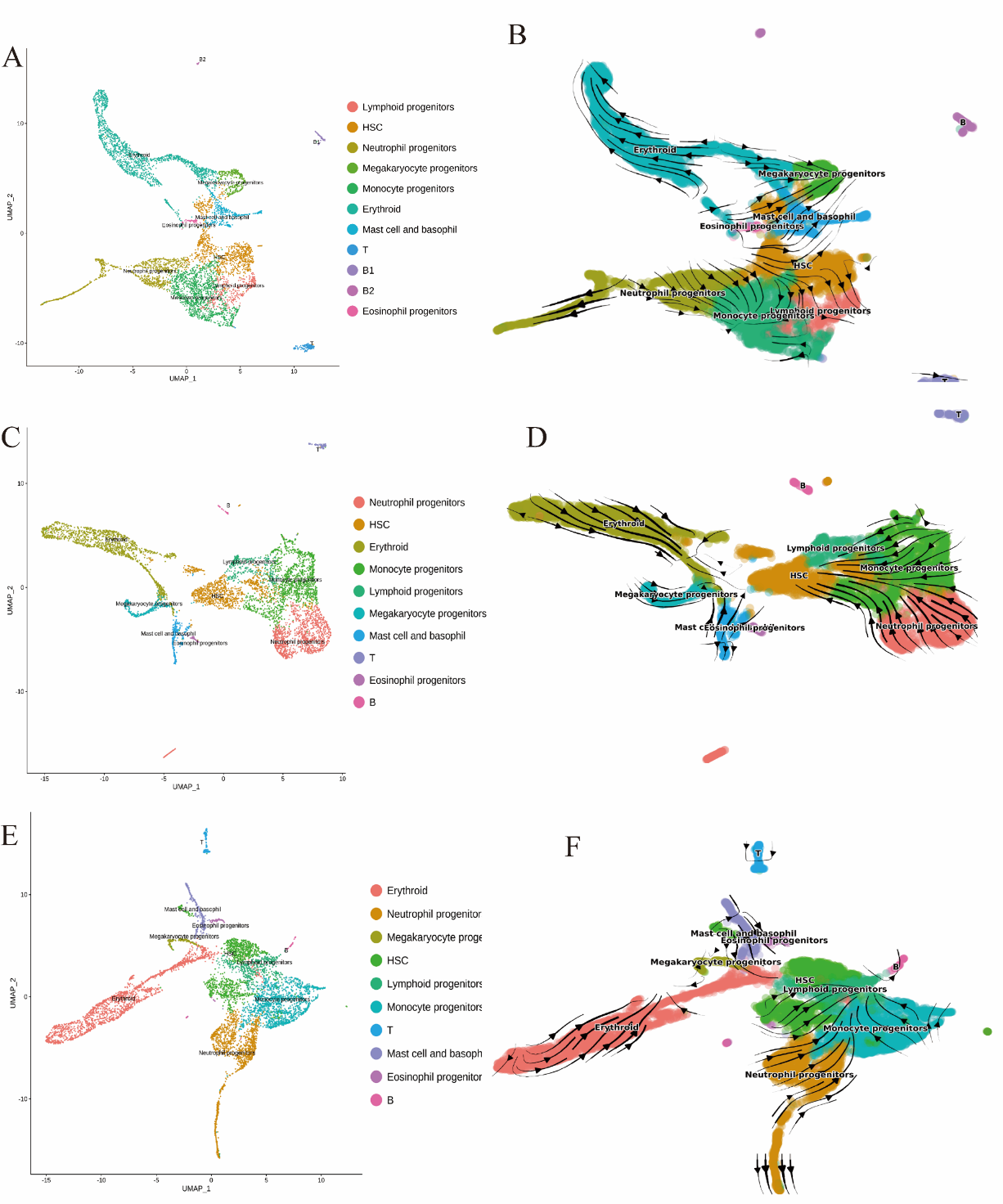


**Supplemental Figure 1. RNA velocity analyses identified the self‑renewal process of HSPCs**

UMAP(A) and RNA velocity (B) analysis indicate a developmental trend of HSPCs towards various cell types in the control group samples.

UMAP(C) and RNA velocity (D) analysis indicate Monocyte and Neutrophil progenitors, show a developmental trend towards differentiating into HSPCs in DimBEn group

UMAP(E) and RNA velocity (F) analysis indicate HSPCs show a developmental trend towards differentiating into lymphoid progenitor cells in DimBEn+CF group.


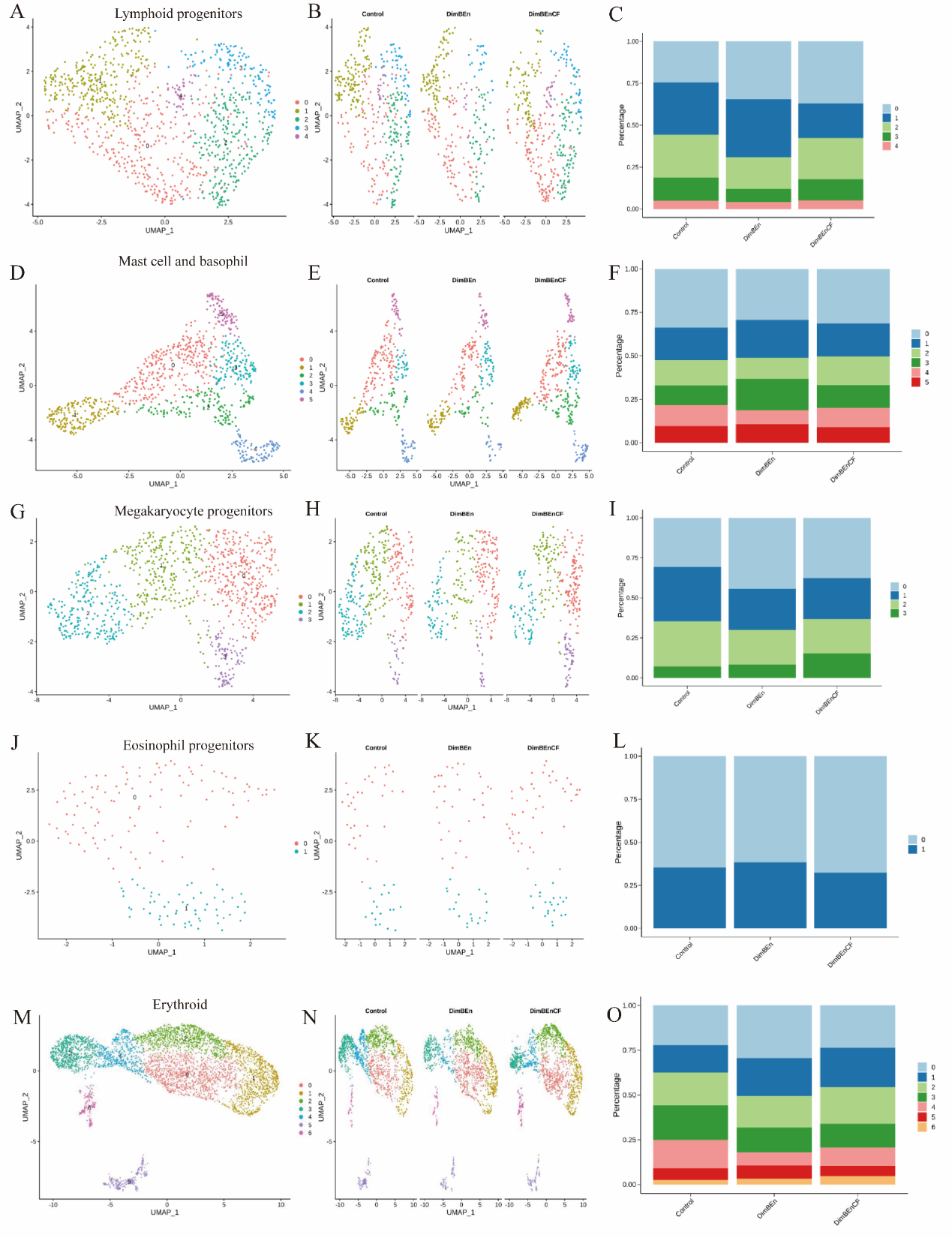


**Supplemental Figure 2. Sub-clusters are involved in the Xylene toxicity in BM.**

UMAP analysis showed that other subgroups in HSPCs.

A. Overview of subcluster of lymphoid progenitors. B. The subcluster of lymphoid progenitors in different sample. C. Bar chart of cell subpopulation proportion between 3 samples;

D. Overview of subcluster of mast cell and basophil cells. E. The subcluster of mast cell and basophil cells in different sample. F. Bar chart of cell subpopulation proportion between 3 samples;

G. Overview of subcluster of megakaryocyte progenitors . H. The subcluster of megakaryocyte progenitors in different sample. I. Bar chart of cell subpopulation proportion between 3 samples;

J. Overview of subcluster of lymphoid progenitors. K. The subcluster of lymphoid progenitors in different sample. L. Bar chart of cell subpopulation proportion between 3 samples;

M. Overview of subcluster of erythroid . N. The subcluster of erythroid in different sample. O. Bar chart of cell subpopulation proportion between 3 samples;


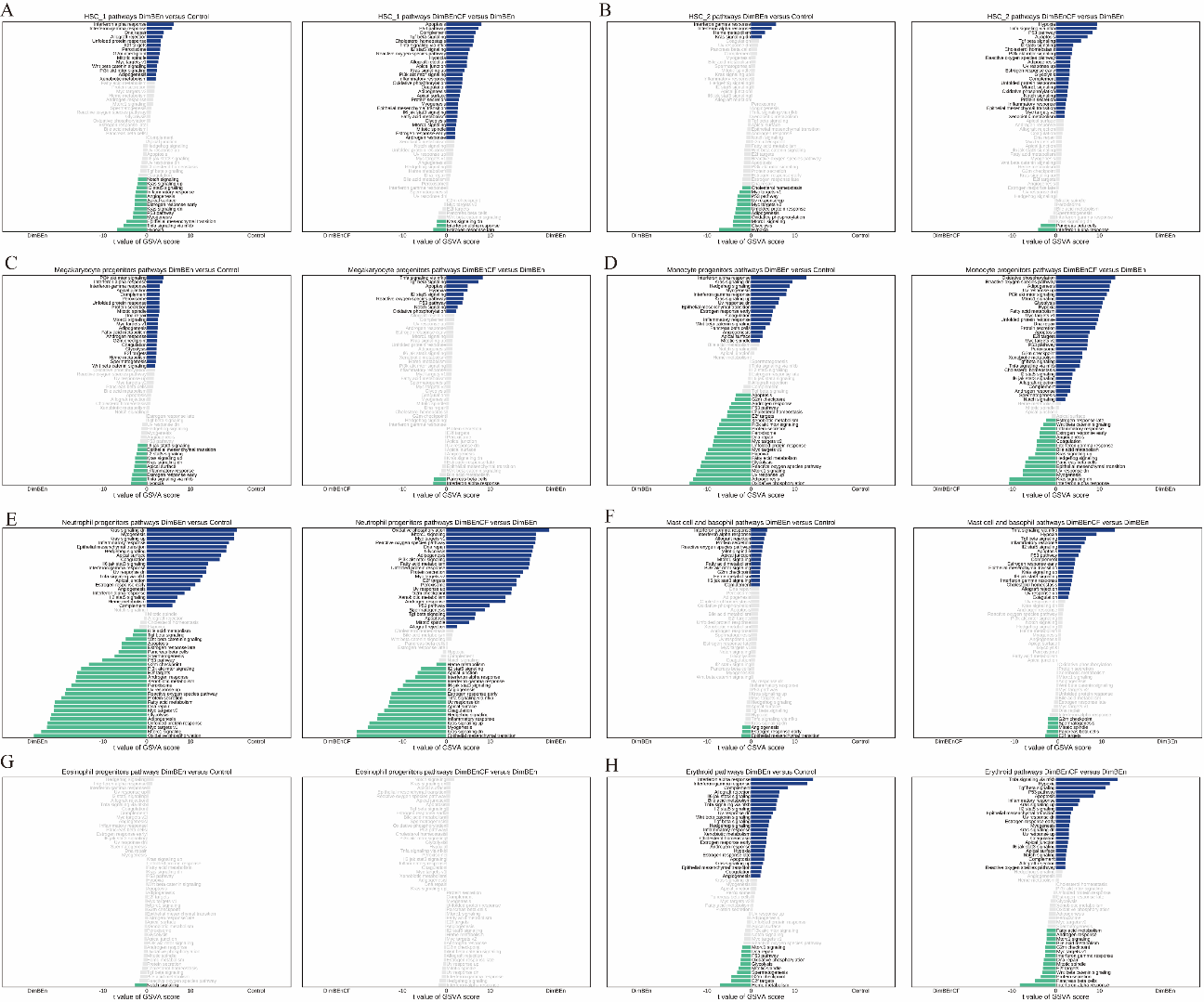


**Supplemental Figure 3. Enrichment analysis of hallmark pathway activities scored per cell by GSVA**

The y-axis represents various gene sets (shown here is the HALLMARK pathway), and the x-axis represents the degree of differential expression of each gene set in different groups. Grey color indicates pathways with no significant differences in expression.

A. HSC1; B. HSC2; C. Megakaryocyte progenitors; D. Monocyte progenitors; E. Neutrophil progenitors F. Mast cell and basophil; G. Eosinophil progenitors; H. Erythroid


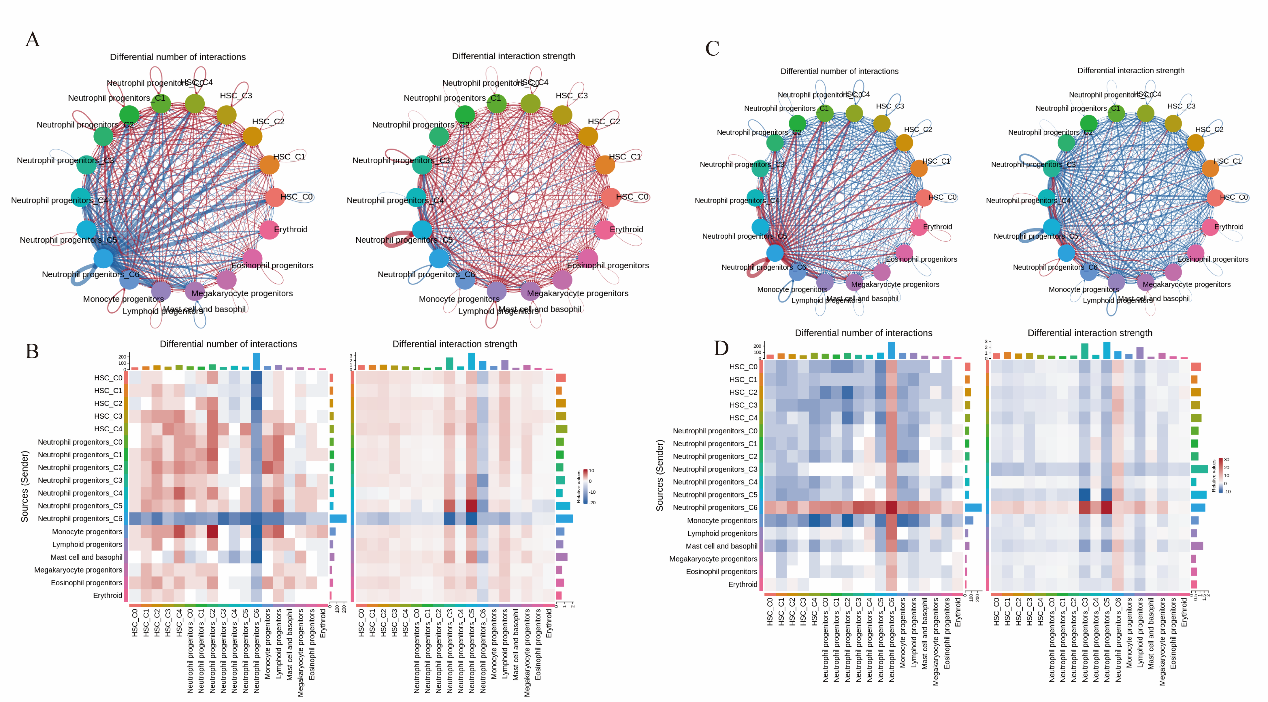


**Supplemental Figure 4. Assessing cell–cell interactions occurring in CF protective xylene induced BM toxicity**

A circular plot can be used to visualize the differences in the number or strength of interactions in the cell-cell communication network between two datasets, where red (or blue) colored edges represent increased (or decreased) signals in the second dataset compared to the first. In the figure (A,C) , red indicates enhanced signaling in the DimBEn group.

A heatmap to display the differences in the number or strength of interactions in more detail. The colored bars at the top represent the total sum of column values (incoming signals) displayed in the heatmap. The colored bars on the right represent the total sum of row values (outgoing signals). In the color bar, red (or blue) indicates increased (or decreased) signals in the second dataset compared to the first. In the figure(B,D), red indicates enhanced signaling in the DimBEn group.


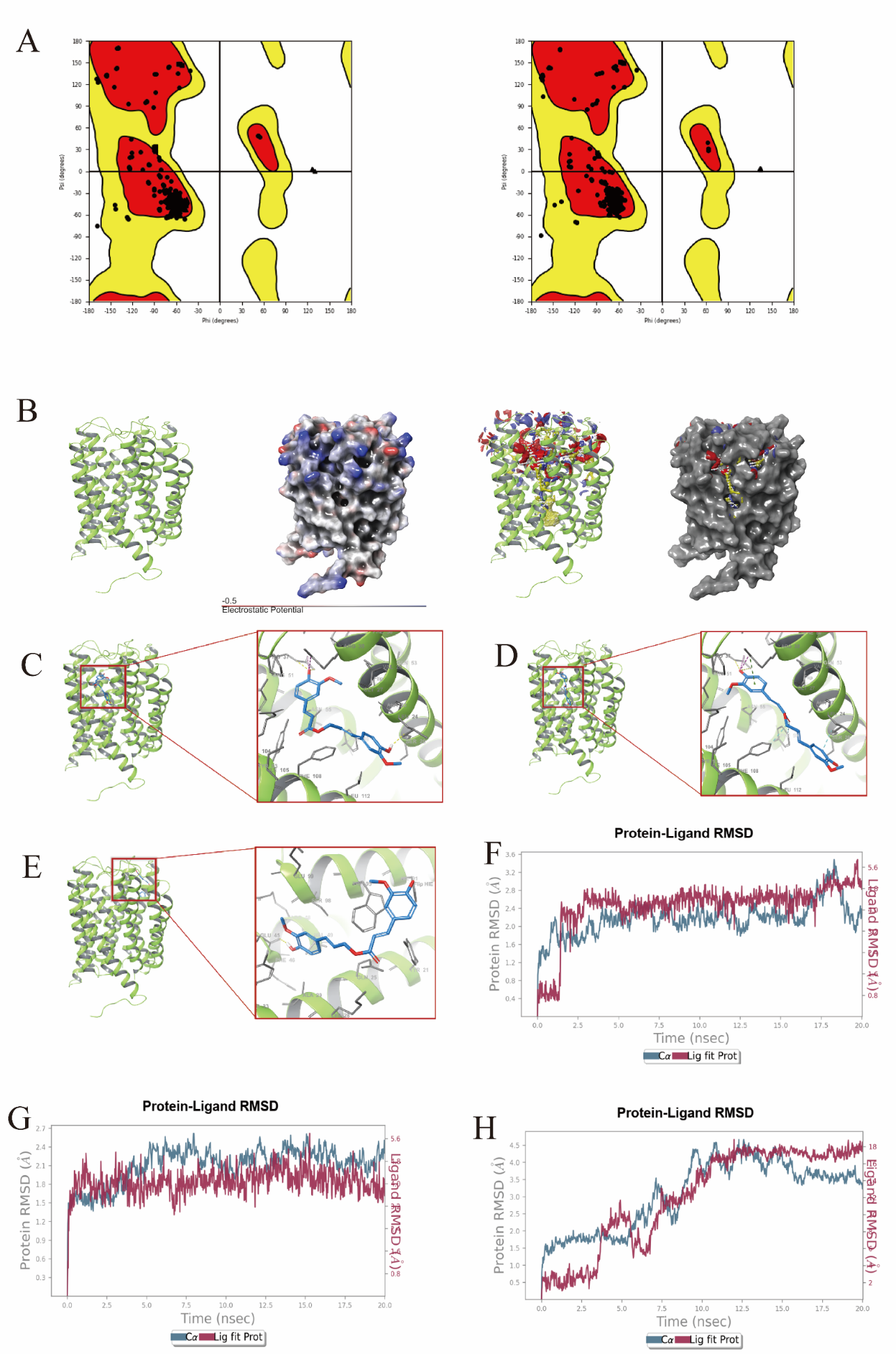


**Supplemental Figure 5. Molecular simulation docking predicted the binding sites between CF and Mgst2.**

Ramachandran Plot plot of the status of amino acid residues before (A, left) and after (A, right) protein structure optimization.

(B) Optimized Mgst2 protein secondary structure and surface morphology; Scanning the position of the possible compound-binding pocket on the Mgst2 protein by the algorithm, Mareling areas that can bind compounds; The presence of multiple compound binding sites at the surface and center of the Mgst2 protein, respectively, These sites will be used for downstream analog docking operations; represent the compound-binding groove structures on the protein surface; White dot: the compound binding pocket region, Red area: H-bond accepter (hydrogen-bond receptor), Blue area: H-bond donor (hydrogen bond donor), Yellow area: the hydrophobic domain (the hydrophobic region). The first (C), second (D) and third (E) conformation and bonding relationship of compound CF binding to Mgst2 protein. RMSD values (A˚ ) of CF alone and CF-peptide1(F), CF-peptide2(I) and CF-peptide3 (J) complex in MD simulation.


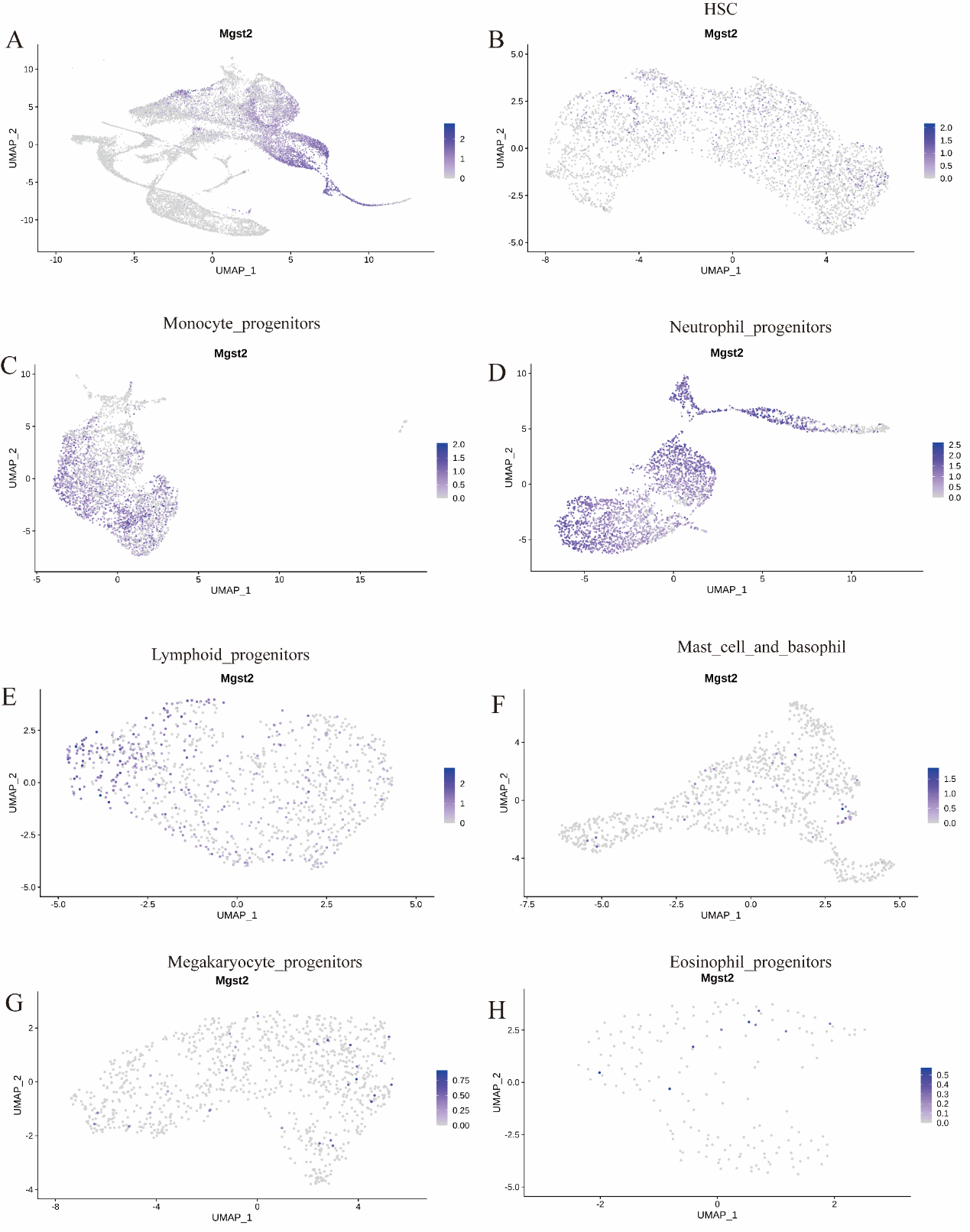


**Supplemental Figure 6. Mgst2 expression in different cell type of HSPCs.**

A.UMAP analysis showed the Mgst2 expression in all cell types

B. UMAP analysis showed the Mgst2 expression in HSC; C. Monocyte progenitors ;D. Neutrophil progenitors ; E. Lymphoid progenitors ;F. Mast cell and basophil ;G. Megakaryocyte progenitors ; H. Lymphoid ; I. Erythroid
